# MicroRNA miR-24-3p reduces DNA damage responses, apoptosis, and susceptibility to chronic obstructive pulmonary disease

**DOI:** 10.1172/jci.insight.134218

**Published:** 2021-01-25

**Authors:** Jessica Nouws, Feng Wan, Eric Finnemore, Willy Roque, So-Jin Kim, Isabel Bazan, Chuan-xing Li, C. Magnus Skold, Qile Dai, Xiting Yan, Maurizio Chioccioli, Veronique Neumeister, Clemente J. Britto, Joann Sweasy, Ranjit Bindra, Åsa M. Wheelock, Jose L. Gomez, Naftali Kaminski, Patty J. Lee, Maor Sauler

**Affiliations:** 1 Section of Pulmonary, Critical Care, and Sleep Medicine, Department of Internal Medicine, Yale University School of Medicine, New Haven, Connecticut, USA.; 2 Department of Anatomy, Beijing University of Chinese Medicine, Beijing, China.; 3 Department of Internal Medicine, Rutgers New Jersey Medical School, Newark, New Jersey, USA.; 4 Division of Respiratory Medicine and Allergy, Department of Medicine, and Center for Molecular Medicine, Karolinska Institutet and Karolinska University Hospital, Stockholm, Sweden.; 5 Department of Biostatistics, Yale School of Public Health, New Haven, Connecticut, USA.; 6 Department of Pathology, Yale University School of Medicine, New Haven, Connecticut, USA.; 7 Department of Radiation Oncology, University of Arizona College of Medicine, Tucson, Arizona, USA.; 8 Department of Therapeutic Radiology, Yale University School of Medicine, New Haven, Connecticut, USA.; 9 Section of Pulmonary, Allergy, and Critical Care Medicine, Department of Internal Medicine, Duke University School of Medicine, Durham, North Carolina, USA.

**Keywords:** Pulmonology, Apoptosis, COPD, DNA repair

## Abstract

The pathogenesis of chronic obstructive pulmonary disease (COPD) involves aberrant responses to cellular stress caused by chronic cigarette smoke (CS) exposure. However, not all smokers develop COPD and the critical mechanisms that regulate cellular stress responses to increase COPD susceptibility are not understood. Because microRNAs are well-known regulators of cellular stress responses, we evaluated microRNA expression arrays performed on distal parenchymal lung tissue samples from 172 subjects with and without COPD. We identified miR-24-3p as the microRNA that best correlated with radiographic emphysema and validated this finding in multiple cohorts. In a CS exposure mouse model, inhibition of miR-24-3p increased susceptibility to apoptosis, including alveolar type II epithelial cell apoptosis, and emphysema severity. In lung epithelial cells, miR-24-3p suppressed apoptosis through the BH3-only protein BIM and suppressed homology-directed DNA repair and the DNA repair protein BRCA1. Finally, we found BIM and BRCA1 were increased in COPD lung tissue, and *BIM* and *BRCA1* expression inversely correlated with miR-24-3p. We concluded that miR-24-3p, a regulator of the cellular response to DNA damage, is decreased in COPD, and decreased miR-24-3p increases susceptibility to emphysema through increased BIM and apoptosis.

## Abstract 2

miR-24-3p, a regulator of the DNA damage response, is reduced in chronic obstructive pulmonary disease lung tissue and inhibits susceptibility to epithelial apoptosis and emphysema.

## Graphical abstract

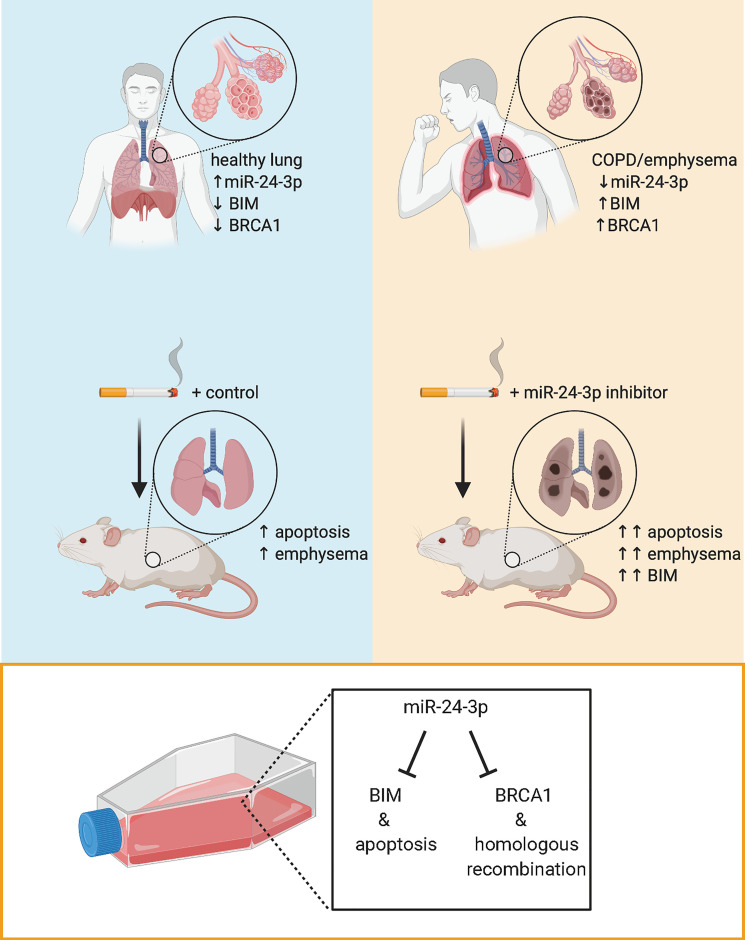

## Introduction

Chronic obstructive pulmonary disease (COPD) is a leading cause of global mortality and is characterized by persistent airflow limitation due to small airway disease and emphysema (1). Chronic exposure to cigarette smoke (CS) is a major risk factor for COPD, and cytotoxic effects of CS contribute to COPD pathogenesis. However, only certain smokers develop clinically significant COPD. Therefore, individual differences in cellular stress responses to CS may be critical determinants of COPD severity, but there remains a limited understanding of the specific cellular responses that protect from or predispose individuals to disease progression (2, 3).

DNA damage is a well-described consequence of CS exposure, and growing evidence from genetic association studies and animal models of disease has suggested an important role for cellular responses to DNA damage in the pathobiology of COPD (4–8). DNA damage occurs in all cells from endogenous (e.g., metabolism) and exogenous (e.g., CS) sources. To promote genomic stability, cells maintain a network of intertwined signaling pathways collectively referred to as the DNA damage response (DDR) (9). The DDR coordinates cell cycle checkpoints, DNA repair, and DNA tolerance pathways. In the setting of severe DNA damage, the DDR activates specific programs such as cellular senescence or apoptosis. However, the degree of DNA damage is not the only determinant of cell fate following DDR activation. Both the capacity to repair DNA and the propensity of cells to undergo apoptosis vary across cell/tissue types, between individuals, and can change with age or disease states (10). In COPD, there is increased activation of the DDR and pathologic consequences of DDR (e.g., apoptosis), even when compared with smokers without COPD (5, 11). While cellular mechanisms that regulate the DDR are likely to be important mediators of COPD susceptibility, there is little understanding of the specific DDR regulators that contribute to COPD pathogenesis.

MicroRNAs are short noncoding RNAs that function as epigenetic regulators of many cellular stress responses, including the DDR (12). In this study, we sought to identify regulators of cellular stress responses that increase susceptibility to COPD and hypothesized that microRNAs regulating the DDR are critical determinants of COPD susceptibility. We analyzed microRNA expression in human lung tissue samples and found miR-24-3p inversely correlated with multiple measurements of disease severity in COPD. To determine the biological relevance of decreased miR-24-3p expression in COPD, we used a mouse model of CS exposure and found inhibition of miR-24-3p increased susceptibility to CS-induced apoptosis and emphysema. In cell culture models, we found miR-24-3p inhibited apoptosis, in part, through inhibition of the proapoptotic BH3-only protein BCL2L11, which is commonly referred to as BIM. We also demonstrated miR-24-3p inhibited the homologous recombination (HR) DNA repair pathway and the HR protein BRCA1. Finally, we found BIM and BRCA1 were increased in lung tissue samples from subjects with COPD, and both *BIM* and *BRCA1* inversely correlated with miR-24-3p expression.

## Results

### miR-24-3p is decreased in COPD.

**Figure 1:**
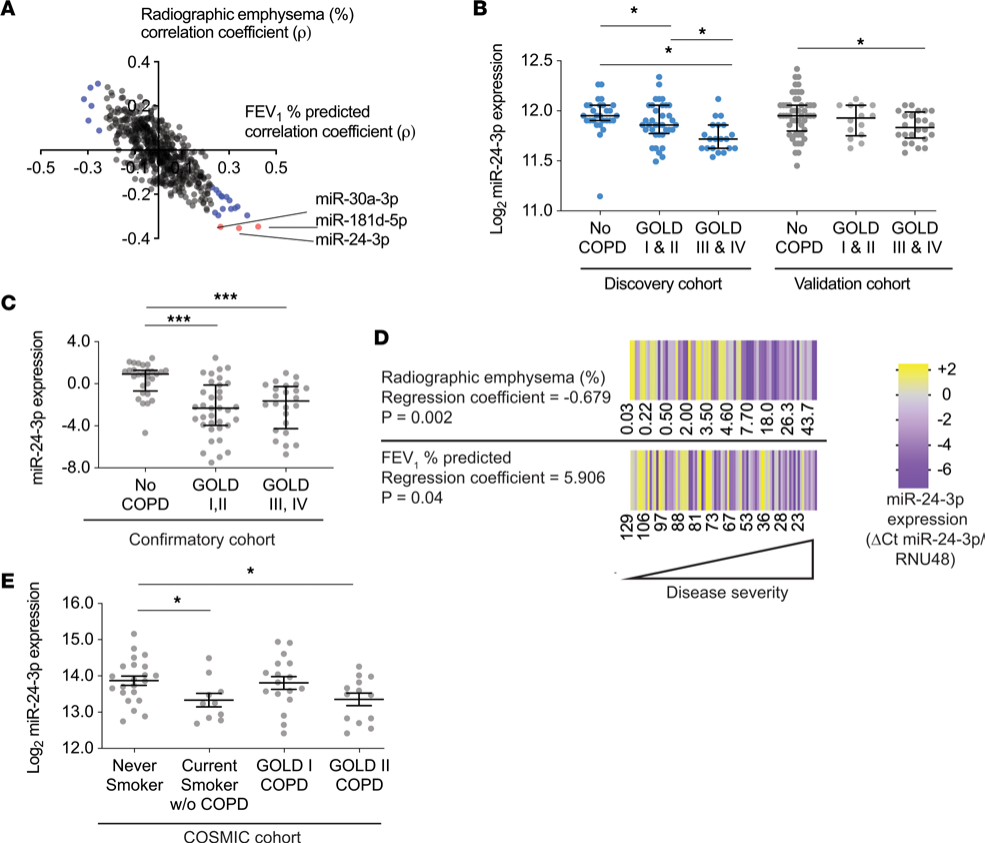
miR-24-3p is decreased in COPD and inversely correlates with disease severity. (**A**) Coefficients of Spearman correlations (ρ) between microRNAs versus percent radiographic emphysema (*y* axis) (*n* = 121) and microRNAs versus FEV_1_ percent predicted (*x* axis) (*n* = 172) in the LGRC cohort. Blue indicates microRNAs correlated with FEV_1_ percent predicted (FDR < 0.05). Red indicates microRNAs correlated with percent radiographic emphysema and FEV_1_ percent predicted (FDR < 0.05). (**B**) Log_2_-transformed microarray expression of miR-24-3p in the discovery and validation LGRC cohorts. discovery cohort: *n* = 28 for No COPD, *n* = 36 for GOLD I & II, *n* = 20 for GOLD III & IV. validation cohort: *n* = 50 for No COPD, *n* = 14 for GOLD I & II, *n* = 24 for GOLD III & IV. (**C**) miR-24-3p expression (ΔCt miR-24-3p/RNU48) measured by RT-PCR in lung tissue samples from the confirmatory cohort. *n* = 28 for No COPD, *n* = 35 for GOLD I & II COPD, and *n* = 24 for GOLD III & IV COPD. (**D**) Heatmap of miR-24-3p expression (ΔCt miR-24-3p/RNU48) measured by RT-PCR in lung tissue samples from the confirmatory cohort versus FEV_1_ percent predicted (*n* = 87) and percent radiographic emphysema (*n* = 75). The regression coefficients and *P* values are adjusted for the effects of age, sex, and smoking status. Yellow denotes increase above the sample median, and purple denotes decrease below the sample median. (**E**) Log_2_-transformed microarray expression of miR-24-3p in airway brushings from the COSMIC cohort. *n* = 22 for never smokers, *n* = 10 for current smokers without COPD, *n* = 17 for current and former smokers with COPD (GOLD I), and *n* = 13 for current and former smokers with COPD (GOLD II). Error bars represent median ± interquartile range (**B** and **C**) or mean ± SEM (**E**). ***P ≤ 0.0001, *P < 0.05, Kruskal-Wallis 1-way ANOVA (**B** and **C**) or ordinary 1-way ANOVA (**E**), correcting for multiple comparisons using the 2-stage linear step-up procedure of Benjamini, Krieger, and Yekutieli.

We analyzed microRNA and mRNA microarray expression profiles of 172 lung parenchymal tissue samples previously performed by the Lung Genomics Research Consortium (LGRC) (13-15), focusing on subjects with and without COPD and excluding subjects with a pathologic diagnosis of pulmonary fibrosis. Table 1 summarizes demographic and clinical characteristics of these 172 subjects. A total of 17 microRNAs positively correlated with FEV_1_ percent predicted, and 6 negatively correlated with FEV_1_ percent predicted (FDR < 0.05) (Figure 1A). Of the 23 correlated microRNAs, 3 microRNAs also negatively correlated with percent radiographic emphysema: miR-181d-3p (*P* = –0.346), miR-551b-3p (*P* = –0.347), and miR-24-3p (*P* = –0.353). All microRNAs correlated with COPD severity measurements are shown in Supplemental Table 1; supplemental material available online with this article; https://doi.org/10.1172/jci.insight.134218DS1

We focused on miR-24-3p because miR-24-3p best correlated with radiographic emphysema and miR-24-3p is highly expressed in the lung (16, 17). We sought to validate our findings by assessing miR-24-3p expression in multiple cohorts. The LGRC cohort was previously divided into discovery and validation cohorts (Supplemental Table 2), and miR-24-3p was decreased in subjects with Global Initiative for Chronic Obstructive Lung Disease (GOLD) III/IV disease in both cohorts (Figure 1B). We then measured miR-24-3p by real-time PCR (RT-PCR) in lung parenchymal tissue samples from subjects in an additional confirmatory cohort. Demographic and clinical characteristics of 87 subjects in this confirmatory cohort are shown in Supplemental Table 3. We found decreased miR-24-3p in patients with GOLD I/II disease (0.36-fold, *P* < 0.0001) and GOLD III/IV (0.27-fold, *P* < 0.0001, Figure 1C). In the confirmatory cohort, we also found miR-24-3p expression positively correlated with FEV_1_ percent predicted (*P* = 0.04) and negatively correlated with percent radiographic emphysema (*P* = 0.002) even after adjusting for age, sex, and smoking status (Figure 1D). We also took note of a previous study by Ezzie et al. in which miR-24-3p was among the top 10 microRNAs decreased in parenchymal lung tissue samples from subjects with COPD (18). While miR-24-3p was not the focus of that study, taken together with the above findings, these data from multiple cohorts suggest miR-24-3p is decreased in parenchymal lung tissue from patients with COPD and miR-24-3p expression inversely correlates with COPD severity.

We then sought to determine if miR-24-3p was decreased in epithelial cells and evaluated miR-24-3p expression in findings from the Karolinska Clinical & Systems Medicine Investigations of Smoking-related Chronic Obstructive Pulmonary Disease (COSMIC), a cross-sectional study in which microarrays were performed on airway brushings obtained via bronchoscopy (19). Compared with subjects in the LGRC cohort, COSMIC had a higher proportion of active smokers and subjects with less severe disease as summarized in Supplemental Table 4. We found miR-24-3p was decreased in epithelial brushings from active smokers without COPD (0.69-fold, *P* = 0.03) and brushings from current and former smokers with GOLD II COPD (0.70-fold, *P* = 0.03, Figure 1E).

### Inhibition of miR-24-3p increases CS-induced emphysema and epithelial apoptosis in mice.

**Figure 2:**
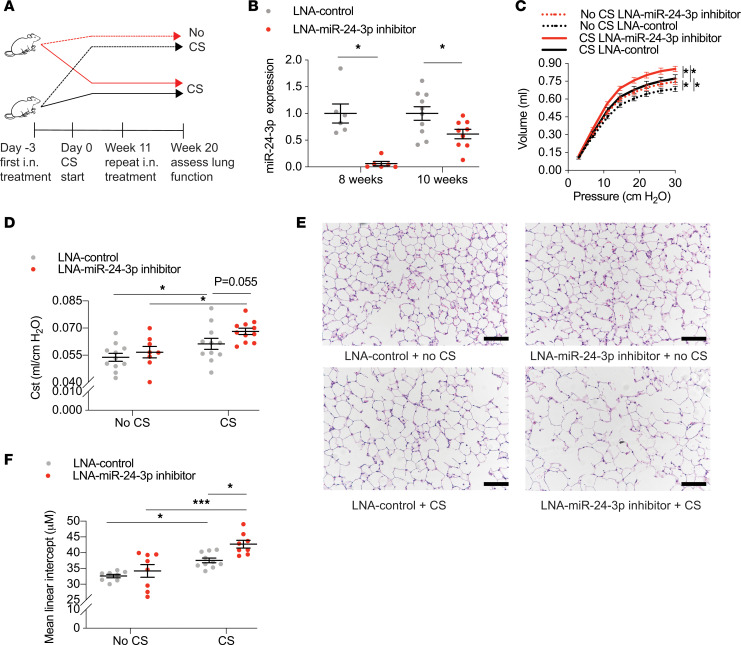
miR-24-3p inhibition increases susceptibility to CS-induced emphysema. (**A**) Study design for mice treated with intranasal (i.n.) LNA–miR-24-3p inhibitor or LNA control ± CS. (**B**) Relative expression of miR-24-3p (ΔΔCt miR-24-3p/snoRNA202) measured by RT-PCR in mouse lungs at 8 or 10 weeks following i.n. administration with LNA-miR-24-3p inhibitor or LNA control (*n* = 6/group for 8 weeks and 9–10/group for 10 weeks). (**C**–**F**) Lung function and histologic assessments of emphysema in mice exposed to CS (*n* = 11/LNA control and *n* = 11/LNA–miR-24-3p inhibitor) and mice exposed to no CS (*n* = 13/LNA control and *n* = 8/LNA–miR-24-3p inhibitor). (**C**) Lung compliance assessed using a flexiVent by the slope of the pressure-volume deflation limb. (**D**) FlexiVent measurements of static lung compliance (Cst). (**E** and **F**) Representative hematoxylin and eosin lung histology and measurements of mean linear intercept (μM) from mice treated with LNA–miR-24-3p inhibitor versus LNA control ± CS. Black scale bar: 200 μm. Error bars represent mean ± SEM. ***P ≤ 0.0001, *P < 0.05, ordinary 1-way ANOVA correcting for multiple comparisons using the 2-stage linear step-up procedure of Benjamini, Krieger, and Yekutieli.

**Figure 3:**
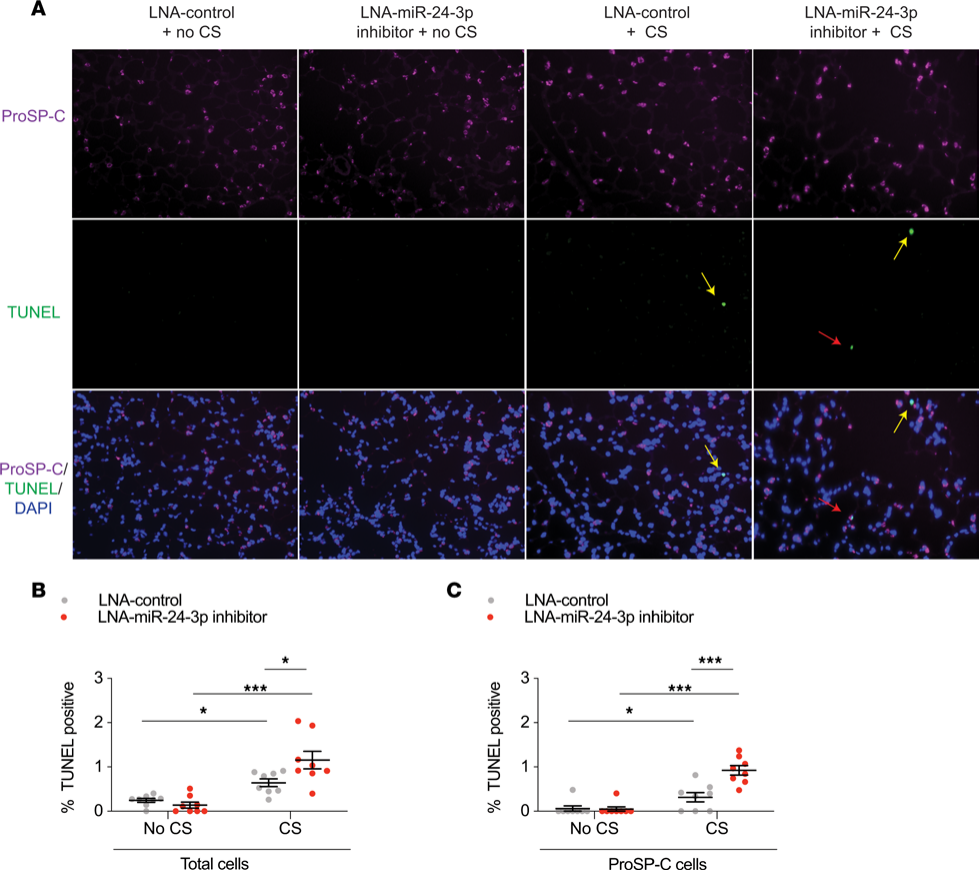
miR-24-3p inhibition increases susceptibility to CS-induced apoptosis. (**A**) Immunofluorescence staining for pro-surfactant protein C (proSP-C) and TUNEL and composite images with DAPI staining in lungs from mice treated with LNA–miR-24-3p inhibitor versus LNA control ± CS exposure. Yellow arrows show TUNEL^+^proSP-C^–^ cells. Red arrows show TUNEL^+^proSP-C^+^ cells. Original magnification, ×20. (**B**) Quantification of TUNEL^+^ cells as a percentage of all cells (*n* = 8/group). (**C**) Quantification of TUNEL^+^proSP-C^+^ cells as a percentage of all proSP-C^+^ cells (*n* = 8/group). Error bars represent mean ± SEM. ***P ≤ 0.0001, *P < 0.05, ordinary 1-way ANOVA correcting for multiple comparisons using the 2-stage linear step-up procedure of Benjamini, Krieger, and Yekutieli.

To determine the consequences of decreased miR-24-3p expression relevant to COPD, we inhibited miR-24-3p in AKR/J mice using a locked nucleic acid (LNA) inhibitor and exposed mice to 20 weeks of CS. Study design is shown (Figure 2A). First, we demonstrated effective inhibition of miR-24-3p 8 weeks (0.061-fold) and 10 weeks (0.62-fold) after i.n. delivery of the miR-24-3p LNA inhibitor (Figure 2B). Mice were then treated with miR-24-3p LNA inhibitor or LNA control and exposed to CS for 20 weeks, which is the approximate length of time required for the development of emphysema in AKR/J mice (20). As expected, mice exposed to CS demonstrated increased lung compliance, airspace enlargement, and apoptosis. Impressively, miR-24-3p inhibition significantly enhanced CS-mediated increases in lung compliance (20.3% vs. 14.8%) (Figure 2, C and D) and airspace enlargement as measured by mean linear intercept (24.8% vs. 15.2%) (Figure 2, E and F). We also demonstrated inhibition of miR-24-3p increased susceptibility to CS-induced apoptosis as suggested by the increase in TUNEL-positive (1.80-fold) (Figure 3, A and B) and cleaved caspase-3–positive cells (1.84-fold) (Supplemental Figure 1). Notably, we identified a marked increase in apoptosis among alveolar type II epithelial cell (AECII) cells expressing proSP-C (2.92-fold; Figure 3, A and C). Collectively, these findings suggest inhibition of miR-24-3p increases susceptibility to emphysema and apoptosis, including a specific increase in AECII susceptibility to apoptosis.

### miR-24-3p inhibits apoptosis by targeting BIM.

**Figure 4:**
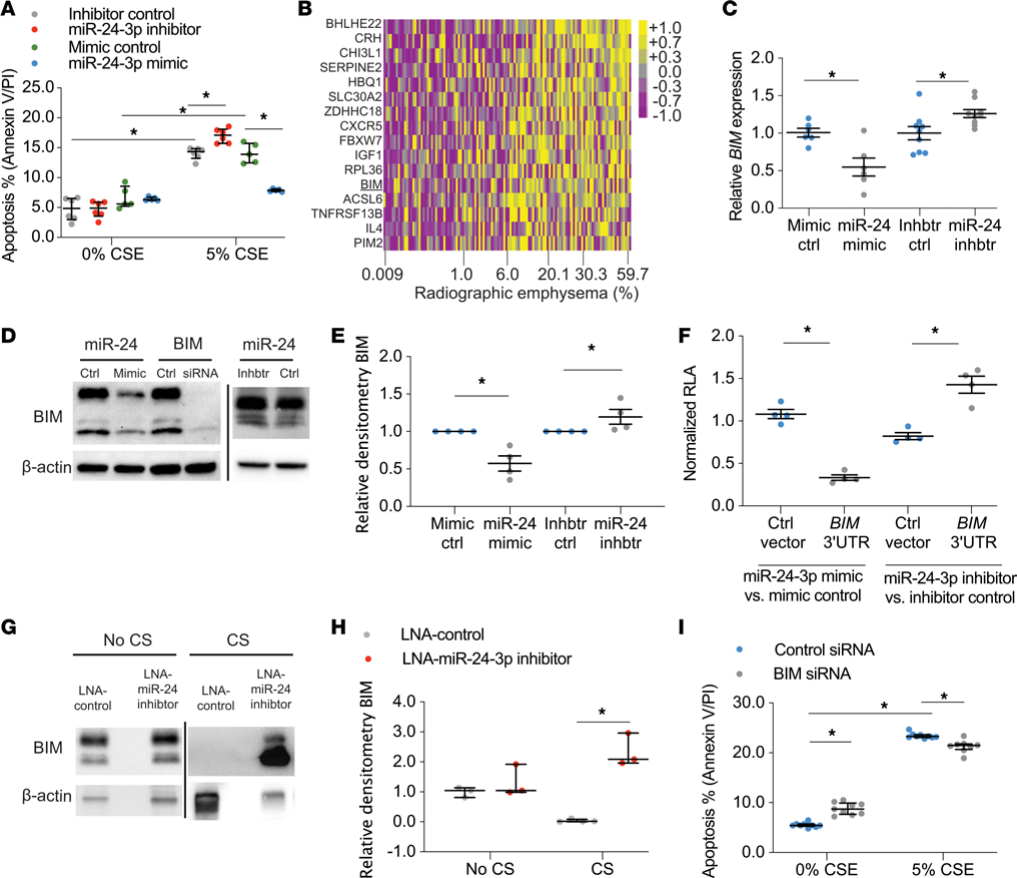
miR-24-3p inhibits apoptosis through BIM. (**A**) Percentage apoptotic cells determined by flow cytometry for annexin V/PI in miR-24-3p mimic versus mimic control (*n* = 5/group) and miR-24-3p inhibitor versus inhibitor control (*n* = 6/group) transfected primary HAECs exposed to 0% or 5% CSE. (**B**) Heatmap of miR-24-3p target genes’ *z* scores, as measured by microarray expression in the LGRC cohort, correlated with percent radiographic emphysema (Spearman ρ, FDR < 0.05) (*n* = 121 subjects). Yellow denotes increase above sample median, and purple denotes decrease below sample median. (**C**) *BIM* expression (ΔΔCt *BIM*/*18S*) measured by RT-PCR in BEAS-2B cells treated with miR-24-3p mimic versus mimic control (*n* = 6/group) and miR-24-3p inhibitor versus inhibitor control (*n* = 9/group). (**D** and **E**) Sample immunoblotting and relative densitometry of BIM/β-actin in BEAS-2B cells treated with miR-24-3p mimic versus mimic control or miR-24-3p inhibitor versus inhibitor control (*n* = 4/group). Sample immunoblotting includes siRNA against *BIM* and siRNA control. (**F**) Relative luciferase activity (RLA) (firefly luciferase/Renilla luciferase) normalized as a ratio of miR-24-3p mimic versus mimic control in BEAS-2B or miR-24-3p inhibitor versus inhibitor control cells cotransfected with *BIM* 3′UTR luciferase reporter plasmid or control plasmid (*n* = 4/group). (**G** and **H**) Sample immunoblotting and relative densitometry of BIM/β-actin performed on lung tissue lysates from mice treated with LNA–miR-24-3p inhibitor or LNA control ± exposure to CS (*n* = 3/group for CS and 4/group for no CS). (**I**) Percentage apoptotic cells determined by flow cytometry for annexin V/PI in BEAS-2B cells treated with siRNA against *BIM* or siRNA control and exposed to 0% or 5% CSE (*n* = 9/group). Error bars represent median ± IQR. *P < 0.05, Kruskal-Wallis correcting for multiple comparisons using the 2-stage linear step-up procedure of Benjamini, Krieger, and Yekutieli. See complete unedited blots in the supplemental material.

We then sought to determine the role of miR-24-3p in epithelial cell responses to CS. To modulate miR-24-3p expression, we used miR-24-3p mimics and miR-24-3p inhibitors to overexpress (6.4-fold) and inhibit (0.039-fold) miR-24-3p, respectively (Supplemental Figure 2). Cigarette smoke extract (CSE) was used to model epithelial response to CS. Transfection of primary human airway epithelial cells (HAECs) with miR-24-3p inhibitor increased susceptibility to CSE-mediated apoptosis (1.27-fold), while transfection with miR-24-3p mimic decreased susceptibility to CSE-mediated apoptosis (0.19-fold) (Figure 4A). miR-24-3p also inhibited CSE-mediated apoptosis in BEAS-2B cells and proSP-C–expressing MLE-12 murine cells as assessed by flow cytometric measurements of annexin V/propidium iodide (PI) and caspase-3/7 (Supplemental Figure 3).

To determine the mechanism through which miR-24-3p inhibits apoptosis, we performed immunoblotting for p53 and phosphorylated p53 (Serine15) and found miR-24-3p inhibition of CSE-mediated apoptosis was p53 independent (Supplemental Figure 4). To determine the p53-independent mechanism via which miR-24-3p inhibits apoptosis, we reanalyzed the 172-patient LGRC data set to identify miR-24-3p target genes correlated with disease severity. We identified 1417 genes inversely correlated with miR-24-3p expression, of which 57 were putative miR-24-3p targets as predicted by TargetScan, miRDB, or mirTarbase. Of these 57 genes, 16 genes positively correlated with percent radiographic emphysema (FDR < 0.05) (Figure 4B), including BIM (21). In order to determine if miR-24-3p regulates BIM in epithelial cells, we transfected BEAS-2B cells with miR-24-3p mimic, miR-24-3p inhibitor, and respective controls. miR-24-3p mimic decreased *BIM* mRNA (0.55-fold) and BIM protein (0.58-fold), while miR-24-3p inhibitor increased *BIM* mRNA (1.26-fold) and BIM protein (1.20-fold) (Figure 4, C–E). Notably, miR-24-3p mimic decreased all 3 isoforms of BIM (BIM EL, BIM L, and BIM S) (Figure 4D). We also found that transfection of miR-24-3p decreased BIM in MLE-12 cells (0.22-fold) (Supplemental Figure 5). We transfected BEAS-2B cells with an expression vector containing the 3′untranslated region (3′UTR) of *BIM* upstream of the firefly luciferase gene and demonstrated that miR-24-3p targets the 3′UTR of *BIM* for degradation (Figure 4F). We also found increased BIM in immunoblots of lung tissue lysates from mice treated with LNA–miR-24-3p inhibitor and exposed to CS (63.6-fold) (Figure 4, G and H), thereby confirming miR-24-3p modulates BIM in vivo. Finally, we sought to determine the role of BIM in CSE-mediated epithelial apoptosis. After demonstrating effective inhibition of BIM with siRNA (Supplemental Figure 6), we found *BIM* inhibition decreased CSE-mediated apoptosis in BEAS-2B cells as assessed by flow cytometric measurements of annexin V/PI (0.91-fold) and caspase-3/7 (0.86-fold) (Figure 4I and Supplemental Figure 7), albeit to a lesser extent than transfection with miR-24-3p mimic. Collectively, these data suggest miR-24-3p inhibits CSE-mediated epithelial apoptosis, in part, through BIM.

### miR-24-3p inhibits homology-directed DNA repair and BRCA1.

**Figure 5:**
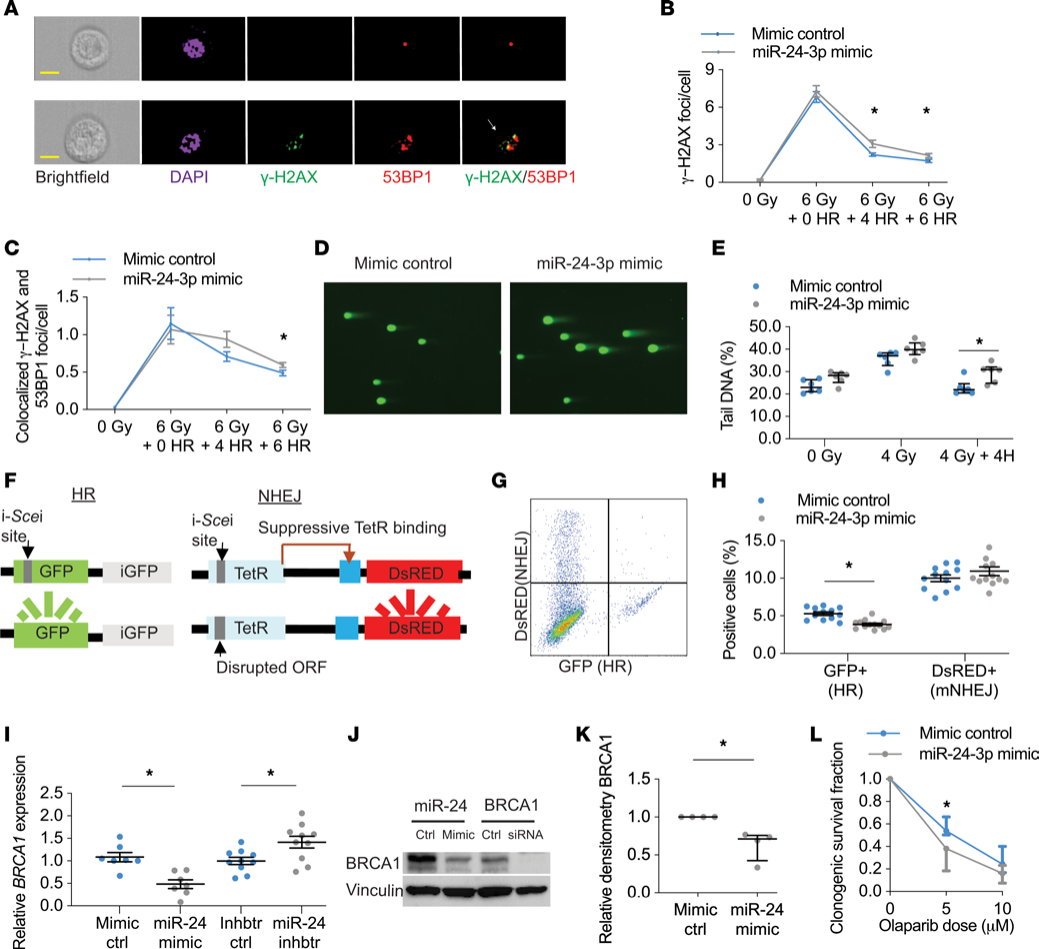
miR-24-3p inhibits HR and BRCA1. (**A**) Imaging flow cytometry of cells exposed to 0 or 6 Gy of IR. Representative images of DAPI, γ-H2AX, and 53BP1 immunofluorescence staining. Colocalized γ-H2AX/53BP1 foci (yellow) are shown (arrow). Yellow scale bar: 10 μm. (**B** and **C**) Primary HAECs exposed to 0 Gy or 6 Gy with 0–6 hours (H) recovery (*n* = 7/group). (**D** and **E**) Comet assay in BEAS-2B cells exposed to 0 Gy, 4 Gy, or 4 Gy with 4H recovery. Percentage tail DNA reflects DNA damage (*n* = 6/group), with sample images following 4 Gy with 4H of recovery (original magnification, ×20). (**F**) Schema of DNA reporter cell assay with 2 integrated loci for measuring HR and mutagenic nonhomologous end joining (mNHEJ). (**G**) Representative flow cytometry demonstrating DsRED^+^ (mNHEJ) and GFP^+^ (HR) expression. (**H**) DNA reporter cells transfected with miR-24-3p mimic (*n* = 13/group) versus mimic control (*n* = 12/group). (**I**) *BRCA1* expression (ΔΔCt of *BRCA1*/*18S*) measured by RT-PCR in BEAS-2B cells treated with miR-24-3p mimic versus mimic control (*n* = 7/group) and miR-24-3p inhibitor versus inhibitor control (*n* = 10/group). (**J** and **K**) Sample immunoblotting and relative densitometry of BRCA1/Vinculin in BEAS-2B cells treated with miR-24-3p mimic versus mimic control (*n* = 4/group). Sample immunoblotting includes siRNA against *BRCA1* and siRNA control. (**L**) BEAS-2B cells transfected with miR-24-3p mimic versus mimic control and treated with olaparib at indicated dosages (*n* = 5/group). Error bars represent mean ± SEM (**B**, **C**, **H**, and **I**) or median ± IQR (**E**, **K**, and **L**). *P < 0.05 ordinary 1-way ANOVA (**B**, **C**, **H**, and **I**), Mann-Whitney (**K**), or Kruskal-Wallis (**E** and **L**) correcting for multiple comparisons using the 2-stage linear step-up procedure of Benjamini, Krieger, and Yekutieli. See complete unedited blots in the supplemental material.

To understand miR-24-3p inhibition of apoptosis within the larger context of the DDR, we sought to determine the effects of miR-24-3p on DNA repair. Previous gene ontology enrichment analyses suggested genes involved in the repair of double-strand DNA breaks (DSBs) are overrepresented among miR-24-3p target genes (22). DSBs are deleterious types of DNA damage caused by CS (Supplemental Figure 8). Cells respond to DSBs by phosphorylating histone H2AX (γ-H2AX) and recruiting DNA repair proteins such as p53-binding protein 1 (53BP1) to form γpH2AX foci and colocalized γpH2AX/53BP1 DNA repair foci (9). We used ionizing radiation (IR), a well-known inducer of DSBs, to model the role of miR-24-3p in DSB repair, and imaging flow cytometry for high-throughput and automated detection of these DNA repair foci (Figure 5A and ref. 23). In HAECs, transfection with miR-24-3p mimic inhibited resolution of γ-H2AX foci and colocalized γ-H2AX/53BP1 foci following IR, suggesting miR-24-3p inhibits DSB repair (Figure 5, B and C). We measured DAPI intensity to estimate cell cycle phase (G_1_ and S/G_2_) and determine if cell cycle phase was confounding our observations. We observed no differences in cell cycle phase between HAECs treated with mimic control and miR-24-3p mimic (Supplemental Figure 9), and we observed diminished DSB repair in miR-24-3p–treated cells even after dichotomizing by cell cycle phase (Supplemental Figure 10, A and B). We performed the same experiments in BEAS-2B cells and similarly observed decreased DSB repair following transfection with miR-24-3p (Supplemental Figure 10, C and D). Gating strategy is shown (Supplemental Figure 11).

To confirm miR-24-3p inhibits DSB repair, we used the neutral comet assay and again found miR-24-3p impaired resolution of DSBs in BEAS-2B cells (0.83-fold) (Figure 5, D and E). DSB repair occurs through 2 major DNA repair pathways, HR and the more error-prone nonhomologous end joining (NHEJ). We utilized a reporter cell line that distinguishes between mutagenic NHEJ and HR (Figure 5, F and G) and found miR-24-3p inhibited HR (0.48-fold) but not mutagenic NHEJ (Figure 5H and ref. 24). We did not find any well-characterized HR genes that correlated with miR-24-3p expression in human lung tissue samples. However, a previous study suggested that miR-24-3p inhibits the canonical HR protein BRCA1 (22). Transfection of BEAS-2B cells with miR-24-3p mimic decreased *BRCA1* mRNA (0.45-fold), and transfection with miR-24-3p inhibitor increased *BRCA1* mRNA (1.42-fold) (Figure 5I). Transfection with miR-24-3p mimic also decreased BRCA1 protein (0.63-fold) (Figure 5, J and K). Finally, we wanted to determine if transfection with miR-24-3p mimic induced functional BRCA1 deficiency. BRCA1-deficient cells are sensitive to poly (ADP-ribose) polymerase inhibitors, such as olaparib, via synthetic lethal interactions (25). As expected, transfection with miR-24-3p increased susceptibility of BEAS-2B cells to olaparib as measured by clonogenic survival assay (Figure 5L). These data suggest miR-24-3p inhibits BRCA1 and HR.

### BIM and BRCA1 are increased in human COPD.

**Figure 6:**
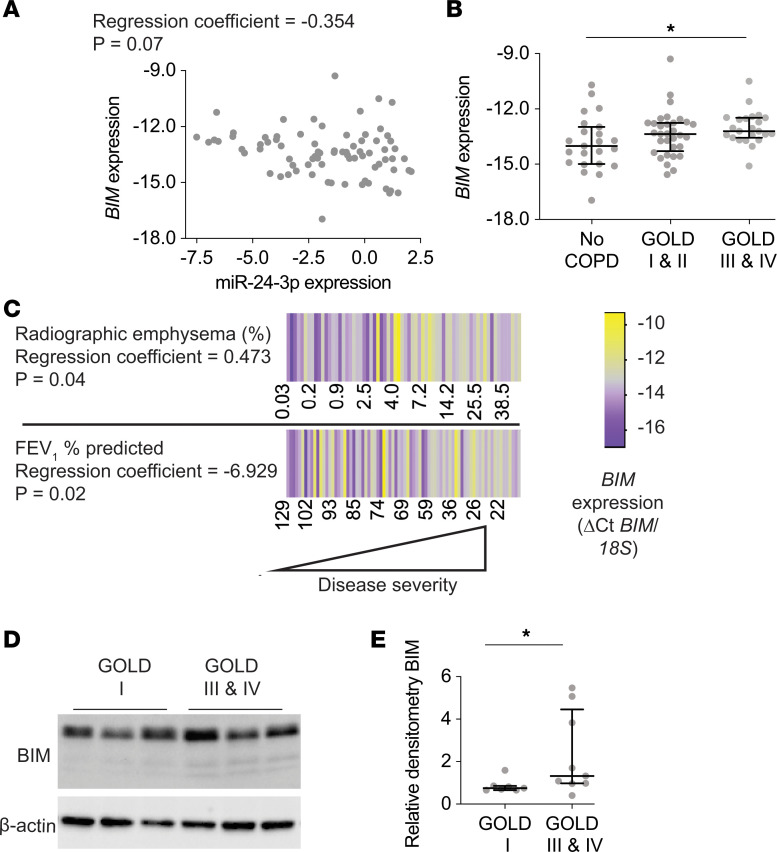
*BIM* expression inversely correlates with miR-24-3p expression and BIM is increased in COPD. (**A**) Correlation of *BIM* expression (ΔCt *BIM*/*18S*) with miR-24-3p expression (ΔCt miR-24-3p/RNU48) measured by RT-PCR in lung tissue samples from the confirmatory cohort (*n* = 78). The regression coefficient and *P* value are adjusted for the effects of age, sex, and smoking. (**B**) *BIM* expression (ΔCt *BIM*/*18S*) measured by RT-PCR in lung tissue samples from the confirmatory cohort. *n* = 23 for no COPD, *n* = 32 for GOLD I & II COPD, and *n* = 23 for GOLD III & IV COPD. (**C**) Heatmap of FEV_1_ percent predicted (*n* = 78) and percent radiographic emphysema (*n* = 68) correlated with *BIM* expression (ΔCt *BIM*/*18S*) measured by RT-PCR in lung tissue samples from the confirmatory cohort. The regression coefficients and *P* values are adjusted for the effects of age, sex, and smoking status. Yellow denotes increase above the sample median, and purple denotes decrease below the sample median. (**D** and **E**) Sample immunoblotting and relative densitometry of BIM/β-actin performed on lung tissue samples from individuals with GOLD I (*n* = 7/group) or GOLD III & IV COPD (*n* = 9/group). Error bars represent median ± IQR. *P < 0.05 Kruskal-Wallis (**B**) or Mann-Whitney (**E**) correcting for multiple comparisons using the 2-stage linear step-up procedure of Benjamini, Krieger, and Yekutieli. See complete unedited blots in the supplemental material.

To determine the relevance of our findings to human disease, we measured BIM in human lung tissue samples. RT-PCR measurement of BIM in the confirmatory cohort demonstrated *BIM* mRNA inversely correlates with miR-24-3p expression, although after adjusting for age, sex, and smoking status, this correlation did not reach statistical significance at *P* < 0.05 (*P* = 0.07) (Figure 6A). We found *BIM* mRNA was increased in patients with GOLD III/IV COPD (1.74-fold, *P* = 0.009) (Figure 6B). We also found *BIM* mRNA inversely correlated with FEV_1_ percent predicted (*P* = 0.02), and *BIM* mRNA correlated with percent radiographic emphysema (*P* = 0.04), even after adjusting for age, sex, and smoking status (Figure 6C). We performed immunoblotting for BIM on lysates of frozen lung tissue samples. Demographic and clinical characteristics of subjects are shown (Supplemental Table 5). We found increased BIM in subjects with GOLD IV COPD compared with GOLD I COPD (1.77-fold) (Figure 6, D and E).

**Figure 7:**
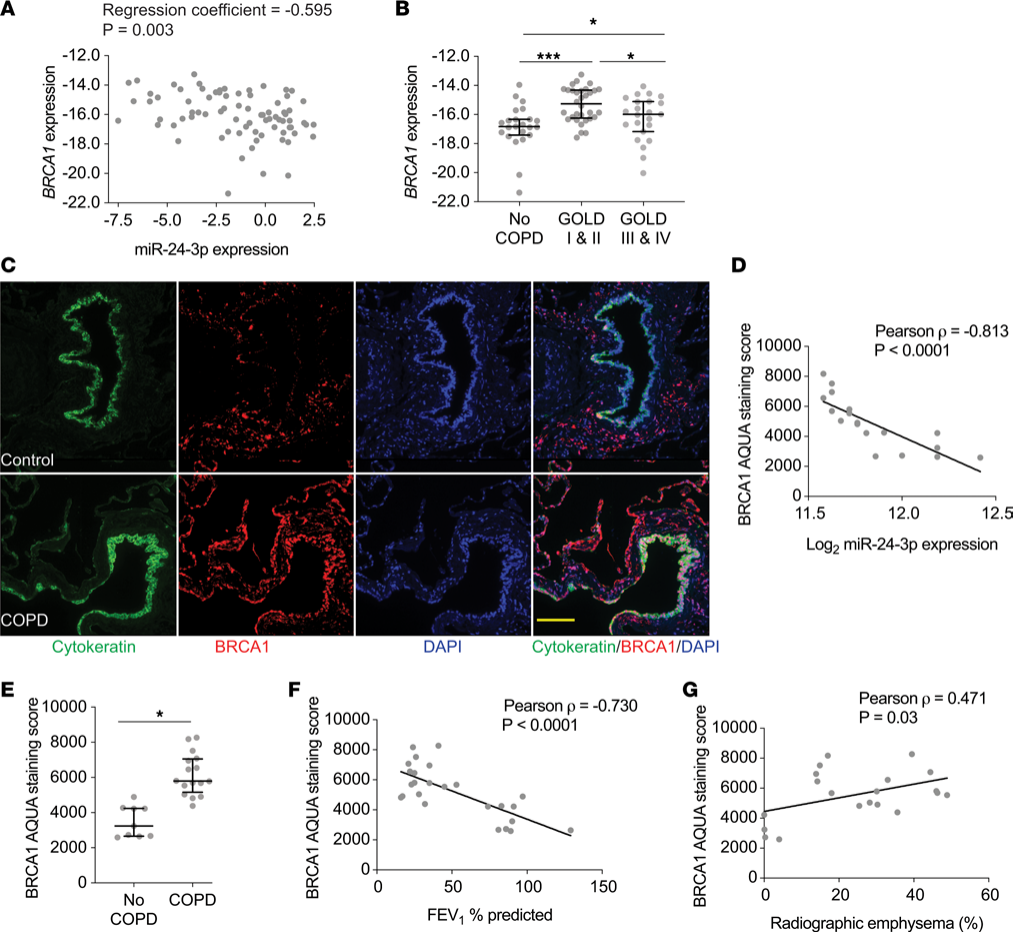
*BRCA1* expression inversely correlates with miR-24-3p expression and BRCA1 is increased in COPD. (**A**) Correlation of *BRCA1* expression (ΔCt *BRCA1*/*18S*) and miR-24-3p expression (ΔCt miR-24-3p/RNU48) measured by RT-PCR in lung tissue samples from the confirmatory cohort (*n* = 78). The regression coefficients and *P* values are adjusted for the effects of age, sex, and smoking status. (**B**) *BRCA1* expression (ΔCt *BRCA1*/*18S*) measured by RT-PCR in lung tissue samples from the confirmatory cohort. *n* = 23 for no COPD, *n* = 32 for GOLD I & II COPD, and *n* = 23 for GOLD II & IV COPD. (**C**) Representative images showing in situ detection of BRCA1, cytokeratin, and DAPI nuclear stain. BRCA1 staining intensity within the image mask generated from the cytokeratin and DAPI-positive staining was used to generate a quantitative score of BRCA1 staining using automated quantitative analysis (AQUA). Yellow scale bar: 50 μm. (**D**) Pearson correlation between miR-24-3p expression and BRCA1 AQUA staining scores (*n* = 19). (**E**) BRCA1 AQUA staining scores from control (*n* = 9/group) and COPD subjects (*n* = 16/group). (**F**) Pearson correlation between FEV_1_ percent predicted and BRCA1 AQUA staining scores (*n* = 25). (**G**) Pearson correlation between percent radiographic emphysema and BRCA1 AQUA staining scores (*n* = 21). Error bars represent median ± IQR (**B**) or mean ± SEM (**E**). ***P ≤ 0.0001, *P < 0.05 Kruskal-Wallis (**B**) or Student’s *t* test (**E**) correcting for multiple comparisons using the 2-stage linear step-up procedure of Benjamini, Krieger, and Yekutieli.

We also measured BRCA1 in human lung tissue samples. RT-PCR measurements of *BRCA1* expression in the confirmatory cohort demonstrated *BRCA1* mRNA inversely correlated with miR-24-3p expression (*P* = 0.003), even after adjusting for age, sex, and smoking status (Figure 7A). We found increased *BRCA1* expression with GOLD I/II COPD (3.58-fold, *P* = 0.0001) and GOLD III/IV COPD (1.79-fold, *P* = 0.04) (Figure 7B), although we also identified a significant decrease in *BRCA1* mRNA expression between GOLD I/II and GOLD III/IV disease (0.60-fold, *P* = 0.01). We then used automated quantitative analysis (AQUA) to quantify BRCA1 protein in human lung tissue. AQUA is a validated method for objectively quantifying protein within defined subcellular compartments (26). Images from lung tissue samples were obtained for 3 channels: DAPI to visualize the nucleus, cytokeratin to identify epithelial cells, and BRCA1 (Figure 7C). Binarization of the DAPI and cytokeratin signal created an image mask of epithelial nuclei, within which we quantified BRCA1 signal intensity. We analyzed BRCA1 in a subset of available paraffin-embedded tissue samples. Demographic and clinical characteristics of subjects are shown (Supplemental Table 6). First, we confirmed BRCA1 measurements by AQUA in the lung were operator independent (Supplemental Figure 12). We found BRCA1 AQUA score inversely correlated with miR-24-3p expression (*P* = –0.813, *P* < 0.0001) (Figure 7D), and we found increased BRCA1 in cytokeratin-positive nuclei from COPD lung tissue samples compared with non-COPD samples (1.76-fold, *P* < 0.0001) (Figure 7E). BRCA1 AQUA scores inversely correlated with FEV_1_ percent predicted (*P* = –0.730, *P* < 0.0001) (Figure 7F) and correlated with percent radiographic emphysema (*P* = 0.471, *P* = 0.03) (Figure 7G). Together, these data suggest *BIM* and *BRCA1* inversely correlate with miR-24-3p expression and BIM and BRCA1 are increased in the lungs of patients with advanced COPD.

## Discussion

In this study, we analyzed microRNA profiles of lung tissue samples from subjects with and without COPD and found miR-24-3p was decreased in COPD and inversely correlated with disease severity (FEV_1_ percent predicted and radiographic emphysema). Decreased miR-24-3p increased susceptibility to CS-mediated emphysema and apoptosis, including AECII apoptosis, in a CS exposure murine model. We also demonstrated that miR-24-3p inhibited apoptosis and HR, and miR-24-3p inhibited the proapoptotic protein BIM and HR protein BRCA1. Finally, we found BIM and BRCA1 are increased in patients with COPD, and *BIM* and *BRCA1* expression inversely correlated with miR-24-3p expression. Together, these findings suggest that decreased miR-24-3p primes the DDR and contributes to COPD severity.

We identified multiple microRNAs that correlated with continuous measurements of airflow obstruction and emphysema (nominal *P* < 0.05) including COPD-associated microRNAs, such as miR-218-5p (27), miR-126-3p (8), miR-223-3p (18), miR-199a-5p (28), and multiple members of the miR-181, miR-34, and miR-30 microRNA families (refs. 29–31 and Supplemental Table 1). However, the microRNA that best correlated with radiographic emphysema was miR-24-3p. miR-24-3p is a member of the miR-23-27-24 microRNA family, which is found in 2 genomic loci in humans (22). While the expression of miR-27a and miR-23a correlated with FEV_1_ percent predicted and percent radiographic emphysema, the expression of miR-27b and miR-23b did not. Therefore, decreased miR-24-3p expression may be related to the function of the miR-23-27-24 cluster on chromosome 19p13. Notably, miR-181d is also located within 19p13, and miR-181d was one of the top 3 microRNAs that correlated with radiographic emphysema in our study (Figure 1A). Therefore, this chromosomal region may be important in mediating disease pathogenesis and future studies are warranted.

We found miR-24-3p inhibited apoptosis in part through the proapoptotic BH3-only protein BIM, and BIM was increased in the lungs of patients with COPD. Apoptosis is a key mechanism of COPD pathobiology and required for the development of emphysema in mice (11). The propensity of cells to undergo apoptosis in response to injury (i.e., “apoptosis priming”) varies across tissue and cell types (32). BIM has been demonstrated to promote apoptosis under physiologic and pathophysiologic conditions (21). While BIM is regulated by diverse signaling mechanisms, posttranscriptional regulation of BIM by miR-24-3p contributes to disease pathology in other organ systems, such as ischemic heart disease (33). Very little is known about the role of BIM in COPD, although increased BIM was identified in a murine model of copper deficiency–induced emphysema (34). Our data suggest that decreased miR-24-3p increases BIM, cellular susceptibility to apoptosis, and disease severity in COPD. One notable finding that deserves mention is the increased apoptosis in unstimulated (i.e., no CSE) BEAS-2B cells treated with siRNA against BIM compared with control when measured by annexin V/PI, although not by caspase-3/7 staining. The reason for this increased baseline apoptosis may be due to homeostatic effects of BIM, such as regulation of autophagy (35), although this role for BIM in BEAS-2B cells will require further study.

Intriguingly, our data also show that miR-24-3p suppressed HR repair of DSBs. Previous studies have also shown that miR-24-3p inhibits DNA repair, but these studies have primarily focused on miR-24-3p suppression of H2AX (36). While H2AX may indeed be a target of miR-24-3p, we did not observe reduced γ-H2AX foci or diminished mutagenic NHEJ following treatment of cells with miR-24-3p mimics. However, we did identify a role for miR-24-3p inhibition of HR and the canonical HR protein BRCA1. We also found *BRCA1* mRNA and BRCA1 protein were increased in COPD lung tissue and inversely correlated with miR-24-3p. This upregulation of BRCA1 may seem paradoxical at first. BRCA1 is involved in DNA repair and has antioxidant effects (37). However, increased DDR activity can have pathologic consequences regardless of *functional* DNA repair capacity. For example, the overexpression of single DNA repair proteins can dysregulate complex DNA repair steps (38). BRCA1 can also facilitate stress-induced apoptosis and activate inflammatory GADD45A signaling, which may contribute to COPD pathogenesis (39, 40). An increase in epithelial BRCA1 suggests that decreased miR-24-3p does not increase apoptosis priming alone, but rather decreased miR-24-3p may prime multiple aspects of the DDR. This notion that regulatory elements prime the DDR in COPD is supported by Paschalaki et al., who identified that miR-126-3p is decreased in COPD and miR-126-3p suppresses the DDR protein ataxia telangiectasia mutated (8). Future studies will be necessary to determine the role of specific DDR elements, such as BRCA1, in COPD pathogenesis.

A key strength of this study is the large number of subjects in the LGRC cohort, allowing us to identify microRNAs *correlated* with COPD-relevant features, as opposed to simply being increased or decreased in disease. Categorical definitions of disease severity (e.g., GOLD stage) do not fully address interpatient variability and can both over- and underestimate disease severity (41). Many have suggested that a better approach to understanding COPD pathogenesis is through the evaluation of multiple continuous disease traits, which our large sample size allowed us to do (2, 42). Another key strength of the study is that we found decreased expression of miR-24-3p across multiple cohorts, and the correlations between miR-24-3p and disease severity remained even after adjusting for common covariates.

However, there are also several limitations to this work that deserve mention. One limitation is that we initially identified miR-24-3p expression changes in parenchymal lung tissue, which is composed of many different cell types. While miR-24-3p is not specifically expressed by any one cell type (17), our findings may be biased by cellular composition changes occurring between normal and COPD parenchymal tissue. However, our data also suggest an important role for reduced miR-24-3p specifically in epithelial cells. We identified miR-24-3p expression changes in airway epithelial cell brushings in the COSMIC cohort and an inverse correlation between miR-24-3p expression and BRCA1 staining in cytokeratin-positive epithelial cells. We found miR-24-3p inhibits BIM and apoptosis in both airway and alveolar epithelial cells in vitro. We also found inhibition of miR-24-3p increases susceptibility to AECII apoptosis in vivo, and previous studies have shown AECII apoptosis is sufficient to cause emphysema in mice (43). The collective findings that miR-24-3p is decreased in both the airway epithelium and parenchymal lung tissue and miR-24-3p mediates biological effects on both airway and alveolar epithelial cells, including AECII cells in vivo, strongly suggest a pathologic role for decreased miR-24-3p expression in the distal and alveolar lung epithelium. However, we acknowledge the possibility that airway epithelial changes identified in the COSMIC cohort may not reflect epithelial changes in the lung parenchyma, and therefore the decrease in parenchymal miR-24-3p expression identified in the LGRC cohort may not be caused by epithelial expression changes. Another limitation is that we found miR-24-3p expression was inversely correlated with parenchymal lung tissue disease severity independent of CS exposure in the LGRC cohort, while there was an equal reduction in miR-24-3p in bronchial epithelium of smokers with and without COPD in the COSMIC cohort. We suspect these divergent findings may be due to differences related to the studied populations, including age and duration of CS exposure. It may also suggest temporal differences between upper and lower airway miR-24-3p expression. However, it raises the possibility that reduced epithelial miR-24-3p expression is insufficient to increase susceptibility to COPD. Future studies using conditional knockout mice, laser-capture microscopy, and/or single-cell sequencing technologies will be necessary to fully elucidate the role of miR-24-3p in COPD pathogenesis.

An additional limitation is that microRNAs can target many different proteins and pathways, and the DDR may not be the only miR-24-3p target in COPD pathogenesis. For example, miR-24-3p was shown to inhibit T cell expression of IL-4, and therefore miR-24-3p expression may also modulate airway inflammation to increase susceptibility to COPD (44). Further studies will be necessary to confirm the role of downstream miR-24-3p targets in mediating COPD pathogenesis. Finally, there is inherent selection bias when obtaining tissue samples from COPD patients. Subjects with early-stage or no COPD are likely being evaluated for a radiographic finding concerning for malignancy while samples from subjects with severe disease are commonly taken from explanted lungs. Even though lung tissue samples are obtained at a distance from suspicious lesions, there is always a concern for a “field of cancerization” effect and the potential role for malignancy as a confounding variable (45). However, a previous array-based study of lung tissue from subjects without cancer also demonstrated decreased miR-24-3p expression in COPD (18).

The key implication of our finding is COPD pathobiology may involve exaggerated and compounding cellular stress responses due to microRNA expression changes, such as increased DDRs and a lowered threshold for apoptosis. Future studies will be necessary to determine the therapeutic role of overexpressing miR-24-3p, as well as developing a robust understanding of effectors and regulators of the DDR in COPD. This may lead to the successful identification of therapeutic approaches to treat COPD that minimize pathogenic DDRs such as apoptosis while preserving DNA repair capacity.

## Methods

### LGRC cohort.

We reanalyzed microRNA and mRNA microarray profiles of flash frozen parenchymal lung tissues performed by the LGRC. The samples were obtained from the National Heart, Lung, and Blood Institute–sponsored (NHLBI-sponsored) Lung Tissue Research Consortium (LTRC) (GSE72967, GSE47460). Lung tissue samples were obtained from explanted, resected, or biopsied lung tissue. Methods of tissue procurement, cohort characteristics, and gene expression profiling have been previously described, and further details are provided in Supplemental Methods. We identified 172 paired mRNA and microRNA expression profiles from patients in the LGRC cohort who did not have a pathologic diagnosis of interstitial lung disease and were designated as “control” or “COPD.” As part of the original study design, samples were previously stratified into discovery and validation cohorts.

### COSMIC cohort.

The Karolinska COSMIC cohort (ClinicalTrials.gov ID: NCT02627872) is a cross-sectional study designed for investigating the molecular pathogenesis of COPD. Methods of tissue procurement, cohort characteristics, and gene expression profiling have been previously described, and further details are provided in Supplemental Methods (19, 46). MicroRNA profiling was performed on airway epithelial brushings collected by fiber-optic bronchoscopy in a subset of the Karolinska COSMIC cohort. Subjects included never-smokers, “healthy” smokers with normal lung function, and individuals with COPD (GOLD I & II). Smokers were matched by smoking history (>10 pack-years) and current smoking habits (10 cigarettes/d the past 6 months).

### Confirmatory cohort for RT-PCR.

We measured miR-24-3p by RT-PCR in 92 available LTRC human lung tissue samples made available through the LGRC who did not have a pathologic diagnosis of interstitial lung disease and were designated as “control” or “COPD.” All samples were run in duplicate. There were 5 miR-24-3p measurements excluded due to inadequate RNA concentrations or a coefficient of variation more than 7.5%. Of the remaining 87 samples, 36 subjects overlapped with the above LGRC microarray analysis while 51 subjects were unique. Subsequently, we measured *BIM* and *BRCA1* by RT-PCR in the same samples. Applying the same exclusion criteria, 9 additional BIM and BRCA1 samples were excluded.

### Quantitative RT-PCR.

The TaqMan MicroRNA Reverse Transcription Kit (Thermo Fisher Scientific) was used to measure miR-24-3p and RNU48 (internal control) per the manufacturer’s protocol. Further details for RNA isolation and quantitative RT-PCR assessments for miR-24-3p, RNU48, snoRNA 202, *BIM*, *BRCA1*, and *18S* are described in Supplemental Methods.

### Cell culture and transfection.

BEAS-2B cells (American Type Culture Collection) were cultured in RPMI 1640 supplemented with 10% heat-inactivated fetal bovine serum (FBS). MLE-12 cells (American Type Culture Collection) were cultured in Dulbecco’s modified Eagle medium (DMEM) with 10% FBS. Both BEAS-2B and MLE-12 cell lines were passaged fewer than 30 times. HAECs (Lonza) were cultured in PneumaCult-expansion medium (StemCell Technologies) and used through passage 6. miR-24-3p mimic, mimic control, miR-24-3p inhibitor, inhibitor control, and the siRNA duplex for silencing BRCA1 and its siRNA nontargeting control were obtained from Thermo Fisher Scientific. The RNA duplex for silencing BIM and its siRNA nontargeting control were obtained from Dharmacon. Cells were transfected in 6-well plates using RNAiMAX transfection reagent (Life Technologies, Thermo Fisher Scientific) and OptiMEM media according to manufacturer’s protocols. Cells were harvested or used for experimentation 24–48 hours after transfection.

### CSE.

Mainstream smoke from one 3RF4 research cigarette (University of Kentucky, Lexington, Kentucky, USA) was bubbled via negative pressure through 10 mL of cell culture media in a fume hood for about 5 minutes. CSE was filtered via 0.22 μM filter (MilliporeSigma). The obtained filtrate was considered as 100% CSE. CSE was sterile filtrated, aliquoted, and stored at –80°C. CSE was quickly thawed for usage and diluted in cell type–specific media at indicated concentrations.

### Apoptosis assays.

Flow cytometry for annexin V and PI was performed per the manufacturer’s protocol (BD Biosciences, 556547). Flow cytometry for caspase-3 and caspase-7 along with SYTOX AADvanced Dead Cell Stain was performed using CellEvent Caspase-3/7 Green Flow Cytometry Assay Kit per manufacturer’s protocol (Thermo Fisher Scientific, C10427). Flow cytometry was performed using a Stratedigm flow cytometer (STD-13).

### Immunoblotting analyses.

Human lung tissue samples used for Western blot to assess BIM were obtained through the LTRC. Samples were homogenized in mammalian protein extraction reagent (Thermo Scientific) supplemented with protease and phosphatase inhibitors (Roche). Western blotting was performed with an SDS-PAGE electrophoresis system (Bio-Rad), on a 4%–20% Tris gel (Bio-Rad) in Tris running buffer, blotted to a PVDF membrane (Bio-Rad) and probed with primary antibodies, including rabbit anti–phosphorylated-p53 (Serine15) (1:1000, Cell Signaling Technologies, 9284), rabbit anti-p53 (1:1000, Cell Signaling Technologies, 9282), rabbit anti-BIM (1:1000, Cell Signaling Technologies, 2933), mouse anti-BRCA1 (1:100, Santa Cruz Biotechnology, sc-6954), rabbit anti-Vinculin (1:10,000, Abcam, ab219649), and HRP-conjugated mouse anti–β-actin (1:5000, Santa Cruz Biotechnology, sc-47778). After washing in TBS with 0.01% Tween, blots were incubated with HRP-conjugated anti-rabbit (1:5000, Cell Signaling Technology, 7074) or HRP-conjugated anti-mouse (1:5000, Cell Signaling Technology 7076) where appropriate, and signal was detected by autoradiography with ECL Supersignal West Femto maximum sensitivity substrate (Thermo Fisher Scientific). The detection of bands was carried out on a ChemiDoc MP imaging system (Bio-Rad Laboratories), and densitometry was performed using ImageJ software (NIH).

### 3′UTR luciferase assay.

Cells were transfected with the firefly luciferase-expressing (pEZX-MT05) plasmids containing the 3′UTR of *BIM* or an empty vector (Genecopoeia) and were cotransfected with miR-24-3p mimic, mimic control, miR-24-3p inhibitor, or inhibitor control using Lipofectamine 2000 (Invitrogen, Thermo Fisher Scientific). After 24–48 hours, the cells were harvested, lysed, and analyzed for firefly and Renilla luciferase using Luc-Pair Duo-Luciferase HS Assay Kit expression as described in the manufacturer’s protocol (Genecopoeia).

### Imaging flow cytometry.

Cells were washed in permeabilization buffer (eBioscience, Thermo Fisher Scientific) and stained overnight at 4°C with FITC-conjugated mouse anti γ-H2AX (Serine139) (1:2000, MilliporeSigma, 16-202A) and Alexa Fluor 647–conjugated rabbit anti-53BP1 (1:400, Novusbio, NB100-304). This was followed by staining with DAPI (1:5000) (Thermo Fisher Scientific) for 5 minutes. Cells were then analyzed with AMNIS imaging flow cytometer (MilliporeSigma). At least 1000 cells were captured per condition at original magnification, ×40, with extended depth of field. Data were analyzed using IDEAS 6.2 imaging flow cytometry software (MilliporeSigma) (23). Full details for the gating strategy are described in Supplemental Methods.

### Neutral comet assays.

Neutral comet assays were performed per manufacturer’s protocol (Trevigen). Briefly, cells were trypsinized and washed with PBS. Samples were suspended in LM Agarose (Trevigen). Neutral electrophoresis was conducted at 21 V for 1 hour. Cells were stained with SYTOX Green nucleic acid stain (Thermo Fisher Scientific), and images of at least 75 cells were captured with a with a Nikon Eclipse Ti microscope.

### DSB reporter assay.

The construction and details of the U2OS cell line with 2 chromosomally integrated host cell reactivation assays has been previously described (24). Briefly, a U2OS cell line was constructed that contains 2 chromosomally integrated host cell reactivation assays, one to measure HR and the other to measure mutagenic NHEJ. The functionality of both vectors relies on expression of an i-*Sce*i endonuclease. The i-*Sce*i endonuclease gene is fused to the ligand binding domain of rat glucocorticoid receptor (GR) on the C-terminus so that the absence of the GR ligand Triamcinolone excludes the i-*Sce*i endonuclease from the nuclease and prevents i-*Sce*i cleavage. To further limit activation of the i-*Sce*i endonuclease, the fusion protein contains a destabilizing domain on the N-terminus. Administration of Shield1 blocks the destabilizing effect. The HR site maintains a green fluorescence reporter (GFP) gene that is interrupted with an i-*Sce*i cleavage site. Upon i-*Sce*i cleavage, a functional GFP gene will be expressed if the cell uses a fragmented and inactivated GFP gene (*iGFP*) on the same or sister chromatid as a template for HR. The NHEJ site maintains a red fluorescence reporter (RFP) gene that is basally repressed by TetR with an i-*Sce*i cleavage site at the TetR locus. If the TetR open reading frame is disrupted, due to i-*Sce*i cleavage and free-end joining during NHEJ, then there is derepression of RFP. For experiments, 2 × 10^5^ U2OS cells were seeded in 2 mL per well in a 6-well plate the day preceding transfection. Cells were grown in high-glucose DMEM with l-glutamine containing 10% tetracycline-free FBS (Gibco, Thermo Fisher Scientific; and Takara Bio). The cells were transfected with miR-24-3p mimic or mimic control. Then, 24 hours later, 500 nM Shield1 (Takara Bio) and 100 nM Triamcinolone acetonide (MilliporeSigma) were added, or no vehicle was added in sham experiments. The cells were harvested 3 days after the removal of Triamcinolone acetonide and Shield1 and washed prior to flow cytometric analysis with a Stratedigm flow cytometer (STD-13).

### Olaparib clonogenic assay.

Cells were transfected with miR-24-3p mimic or mimic control. After 24 hours, cells were reseeded and treated with 0 M, 5 μM, and 10 μM concentrations of olaparib (Selleckchem) dissolved in DMSO (MilliporeSigma). Cells were incubated for 5 days and colonies were stained with a mixture of 6.0% glutaraldehyde (MilliporeSigma) and 0.5% crystal violet (Electron Microscopy Sciences). Methanol was added to solubilize the dye. Absorbance at 540 nm was measured using Cytation 3 Cell Imaging Multi-Mode Reader (BioTek).

### AQUA for BRCA1 immunofluorescence.

This technique has been previously described (26). Paraffinized lung tissue samples to assess BRCA1 AQUA score were obtained through the LTRC. Slides were deparaffinized with xylene and rehydrated with ethanol. Antigen retrieval was performed using citrate buffer (pH 6). Nonspecific antigens were blocked with 30-minute incubation in 3% bovine serum albumin in 0.1 mol/L of Tris-buffered saline for 30 minutes at room temperature. Slides were incubated with primary antibodies for pan-cytokeratin antibody (1:100, DAKO, Agilent, Z0622) and BRCA1 at 4°C overnight (1:1000, Abcam, MS110) (antibody validation described previously) (47), followed by incubation with Alexa Fluor 546–conjugated goat anti-rabbit secondary antibody (1:100, Molecular Probes, Thermo Fisher Scientific, A11010) in mouse EnVision reagent (DAKO, Agilent) for 1 hour. Signal was amplified with Cy5-Tyramide (PerkinElmer) for 10 minutes, and then slides were mounted with ProlongGold + DAPI (Life Technologies, Thermo Fisher Scientific). Fluorescent images were obtained with an Olympus AX-51 epifluorescent microscope. Between 18 and 67 fields of view were quantified for each specimen. Integration of the binarized DAPI and cytokeratin signal generates a compartment image mask. AQUA scores were generated by dividing the sum of the BRCA1 pixel intensities, by the area of the target compartment (DAPI/cytokeratin image). Analysis was carried out using the AQUAnalysis software (Genoptix).

### Mice and CS exposure.

Animal studies were conducted in accordance with the NIH *Guide for the Care and Use of Laboratory Animals* (National Academies Press, 2011). AKR/J mice obtained from The Jackson Laboratories were bred in our facility. Both female and male mice (10–12 weeks old) were used for CS exposure. Littermates were randomized to receiving CS or no CS. Mice were exposed in a Teague TE-10 smoking machine (Teague Enterprises) to CS from 3R4F research cigarettes (University of Kentucky). The number of cigarettes was adjusted between 5 and 10 per cycle (9 min/cycle) to meet a concentration of 100 mg/m^3^. Cigarette concentration was checked every week. Each smoldering cigarette was puffed for 2 seconds at a flow rate of 1.05 L/min to provide a standard puff of 35 cm^3^. Mice received 6 hours’ exposure per day, 5 d/wk for 5 months, and were sacrificed the day after last CS exposure. Sample collection, measurements of lung compliance, and morphometric assessments of lungs have been previously described and are further detailed in the Supplemental Methods (48).

### miR-24-3p LNA inhibitor and scramble control.

Custom miR-24-3p LNA inhibitor and scramble LNA control with full phosphorothioate backbones were designed (Exiqon). Mice were anesthetized with isoflurane and 5 mg/kg of either miR-24-3p LNA inhibitor or LNA control in 50 μL PBS was delivered via i.n. administration. Mice received 1 dose and then were randomized to CS exposure or non-CS exposure groups. All mice received a subsequent dose after 10 weeks of exposure.

### Immunofluorescence and immunohistochemistry for apoptosis.

Slides were deparaffinized with xylene and rehydrated with ethanol. Antigen retrieval was performed using Tris-EDTA buffer (pH 9). TUNEL staining was performed using the TUNEL Assay Apoptosis Detection Kit, CF 488A (Biotium), per manufacturer’s protocol. Nonspecific antigens were blocked with DAKO blocking solution (Agilent). Slides were incubated with anti–proSP-C antibody (1:2000, MilliporeSigma, AB3786) overnight at 4°C, followed by incubation with Alexa Fluor 648–conjugated chicken anti-rabbit secondary antibody (1:100, Life Technologies, Thermo Fisher Scientific, A21443) at room temperature for 1 hour and application of Vectashield mounting media with DAPI (Vector Laboratories). Images were captured with a Nikon Eclipse Ti microscope. Automated counting of total cells and proSP-C–positive cells was performed using ImageJ software functions “threshold” and “analyze particles,” with a size threshold of 2 μm^2^ and circularity > 0.1. Immunohistochemistry for cleaved caspase-3 was performed using rabbit anti–cleaved caspase-3 (Asp175) (1:50, R&D Systems, Bio-Techne, MAB836). The slides were deparaffinized and rehydrated with distilled water. They were then placed in EDTA epitope retrieval buffer at 95°C for 30 minutes, then cooled and rinsed, and then placed in TBS with Tween. This is the same solution that was used in subsequent washing steps. Endogenous peroxidase was blocked using 3% hydrogen peroxide, then rinsed. The slides were then rinsed, and antibodies were detected with HRP-conjugated anti-rabbit secondary antibody. DAB was used to identify the reaction. Then the slides were washed and then counterstained in hematoxylin, dehydrated, cleared, and mounted with resinous mounting media. Images were acquired with a Nikon DS-Ri2 microscope.

### Statistics.

We used a 2-tailed Student’s *t* test for 2-group comparisons of normally distributed continuous data and a Mann-Whitney *U* test for 2-group comparisons of non-normally distributed continuous data. A 1-way ANOVA was used for multiple-group comparisons of normally distributed continuous data, and a Kruskal-Wallis test was used for multiple-group comparisons of non-normally distributed continuous data. Corrections for multiple comparisons were made using the 2-stage step-up method of Benjamini, Krieger, and Yekutieli. Normality (Gaussian distribution) of the data sets was tested with D’Agostino-Pearson test or Shapiro-Wilk normality test. Associations between continuous variables were assessed using a Pearson correlation coefficient for normally distributed data and a Spearman correlation coefficient for non-normally distributed data. To determine the correlations between miR-24-3p, *BIM*, and *BRCA1* expression with each other, FEV_1_ percent predicted, or percent emphysema controlling for the effect of age, sex, and smoking status, multiple linear regression models were performed using R statistical software. In these regression models, natural log transformation was performed on emphysema, *BIM*, miR-24-3p, and *BRCA1* expression values due to their highly skewed distributions. In regression models, all continuous explanatory variables were standardized by subtracting the mean and dividing by the standard deviation. Smoking status categorical variables were never-smokers or former smokers. There was only 1 current smoker in the data set, so this value was removed for multivariate models. For all non–array-based studies, a *P* value of less than 0.05 was considered significant. For apoptosis assays involving CSE, we accounted for batch effect by normalizing the difference between experimental groups around the mean of all experimental groups. For array-based studies, an FDR < 0.05 was considered significant, and BRBarray tools was used for analysis (49).

For mouse studies, 28 mice (12 males and 16 females) were treated with miR-24-3p LNA inhibitor, and 28 mice (12 males, and 16 females) were treated with LNA control. All mice were randomly selected among littermates among 4 breeding cages. Mice were then randomized to receive either CS or no CS, stratifying by sex and treatment group. These sample sizes allowed for 80% power to detect a difference of 17.5% between the 2 groups at a statistical significance level of 0.05. Actual sample size differed due to mortality or automated detection of erroneous flexiVent measurements. Final sample size was LNA control + CS (5 males, 6 females) LNA–miR-24-3p inhibitor + CS (6 males, 5 females), LNA control + no CS (6 males, 7 females), and LNA–miR-24-3p inhibitor + no CS (2 males, 6 females).

### Study approval.

All studies on human samples were approved by the Human Investigation Committee at Yale University (1409014689) (2000020056). All human tissue samples obtained directly from the LTRC or through the LGRC were collected after ethical review for the protection of human subjects and were approved by the NHLBI-sponsored LTRC. The COSMIC study was approved by the Stockholm regional ethical board (2006/959-31/1). Written informed consent was received from all participants prior to sample procurement and study inclusion. All animal studies were approved by the Institutional Animal Care and Use Committee, Yale University (07867).

## Author contributions

MS generated the main conceptual ideas and supervised the project. JN, JS, PJL, and MS designed research studies. JN, FW, EF, and SJK conducted experiments. VN conducted experiments and provided oversight for AQUA of BRCA1. RB provided reagents and oversight for DNA reporter cell line experiments. CMS and AMW supervised the COSMIC study. NK supervised data from LGRC. JN, EF, WR, IB, SJK, CL, MC, QD, XY, and JLG contributed to the data analysis. MS wrote the manuscript with critical appraisal from NK, PJL, JS, RB, CJB, and AMW. All authors contributed to the final version of the manuscript. The order of appearance of co–first authors was determined by seniority and relative contribution to the manuscript.

## Supplementary Material

Supplemental data

## Figures and Tables

**Table 1:**
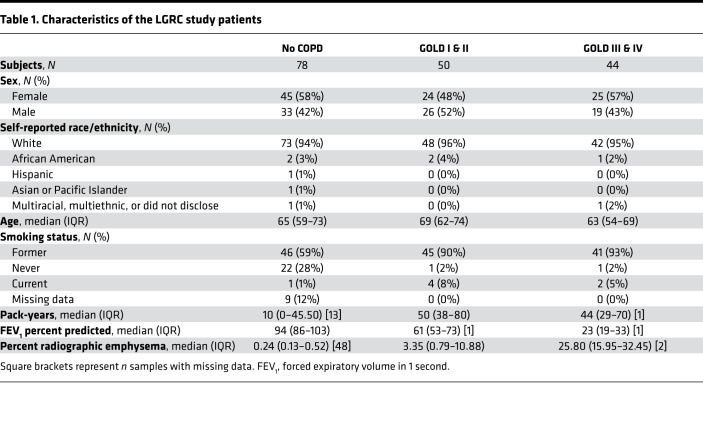
Characteristics of the LGRC study patients

## Version 1 12/08/2020

In-Press Preview

## Version 2 01/25/2021

Electronic publication
