# Supplementary material for: MicroRNA miR-24-3p reduces DNA damage responses, apoptosis, and susceptibility to chronic obstructive pulmonary disease
Source: JCI Insight. 2021 Jan 25;6(2):e134218. doi: 10.1172/jci.insight.134218 (PMC7934877; doi:10.1172/jci.insight.134218)
Supplement: Supplemental data [file jciinsight-6-134218-s057.pdf]

Supplemental Files

Supplemental Tables	Pages 2 – 7
Supplemental Figures	Pages 8 - 14
Supplemental Methods	Pages 15 - 16

Supplemental Table 1 Spearman correlations of microRNAs with FEV_1 percent predicted (*left*) and percent radiographic emphysema (*right*) with a minimum P-value > 0.05. FDR = False Discovery Rate.

Spearman Correlation with FEV1				Spearman Correlation with percent emphysema			
Correlation coefficient	Parametric p-value	FDR	UniqueID	Correlation coefficient	Parametric p-value	FDR	UniqueID
0.424	<1e-07	<1e-07	hsa-miR-181d-5p	-0.353	0.000131	0.0231	hsa-miR-24-3p
0.378	5.00E-07	9.65E-05	hsa-miR-551b-3p	-0.347	0.0001684	0.0231	hsa-miR-30a-5p
0.343	5.40E-06	0.000695	hsa-miR-24-3p	-0.346	0.0001797	0.0231	hsa-miR-181d-5p
0.336	8.30E-06	0.000801	hsa-miR-30d-5p	0.301	0.0011744	0.0967	hsa-miR-199a-5p
0.326	1.64E-05	0.00127	hsa-miR-30b-5p	-0.296	0.001453	0.0967	hsa-miR-551b-3p
-0.316	2.98E-05	0.00192	hsa-miR-21-5p	-0.295	0.0015027	0.0967	hsa-miR-338-3p
0.309	4.44E-05	0.00245	hsa-miR-203-3p	0.285	0.0021706	0.101	has-miR-144-3p
0.302	6.85E-05	0.00331	hsa-miR-30a-5p	0.283	0.0023347	0.101	hsa-miR-4306
-0.297	9.19E-05	0.00394	hsa-miR-148a-3p	-0.283	0.0023656	0.101	hsa-miR-181a-5p
0.29	0.0001345	0.00501	hsa-miR-181c-5p	-0.272	0.0035378	0.123	hsa-miR-331-3p
-0.289	0.0001428	0.00501	hsa-miR-4306	0.268	0.0039881	0.123	hsa-miR-451a
-0.284	0.0001839	0.00592	hsa-miR-21-3p	-0.266	0.004268	0.123	hsa-miR-203-3p
0.274	0.0003161	0.00939	hsa-miR-181b-5p	-0.266	0.0043811	0.123	hsa-miR-181b-5p
0.271	0.0003657	0.0101	hsa-miR-151-3p	-0.265	0.0045259	0.123	hur_6
0.268	0.0004388	0.0113	hsa-miR-30c-2-3p	-0.263	0.0047964	0.123	hsa-miR-30b-5p
0.264	0.00052	0.0125	hsa-miR-30a-3p	0.26	0.0053106	0.128	hsa-miR-199a-3p
0.257	0.0007277	0.0157	hsa-miR-27a-3p	-0.256	0.0060748	0.138	hsa-miR-30d-5p
-0.257	0.0007311	0.0157	hsa-miR-199a-5p	-0.251	0.0072342	0.155	hsa-miR-200c-3p
0.255	0.00081	0.0165	hsa-miR-338-3p	0.248	0.0078927	0.16	hsa-miR-144-3p
-0.252	0.0009592	0.0185	hsa-miR-142-3p	-0.244	0.0089303	0.172	hsa-miR-598-3p
0.246	0.0012341	0.0227	hsa-miR-455-5p	-0.241	0.0100427	0.184	hsa-miR-197-3p
0.241	0.0015924	0.0279	hsa-miR-181a-5p	0.238	0.0108938	0.184	hsa-miR-486-5p
0.229	0.0026854	0.0451	hsa-miR-210-3p	-0.238	0.0109631	0.184	hsa-miR-151a-5p
0.224	0.0033658	0.0541	hsa-miR-598-3p	-0.236	0.0117602	0.184	hsa-miR-181a-3p
0.221	0.003784	0.0584	hsa-miR-4317	0.235	0.0118969	0.184	hsa-miR-223-3p
0.219	0.0041403	0.06	hsa-miR-126-3p	0.231	0.0135586	0.201	hsa-miR-21-3p
-0.219	0.0041947	0.06	hsa-miR-223-3p	-0.23	0.0140969	0.202	hsa-miR-33a-5p
-0.216	0.0048161	0.0664	hsa-miR-144-3p	-0.228	0.014834	0.204	hsa-miR-30a-3p
0.214	0.0050626	0.0674	hsa-miR-498	-0.226	0.0155854	0.207	hsa-miR-126-3p
-0.21	0.0060417	0.0762	hsa-miR-185-5p	-0.223	0.0173123	0.223	hsa-miR-30c-5p
0.21	0.0061181	0.0762	hsa-miR-455-3p	0.217	0.0203321	0.249	hsa-miR-198
0.209	0.0063324	0.0764	hsa-miR-3065-5p	0.217	0.0207598	0.249	hsa-miR-483-5p
-0.207	0.0068105	0.0774	hsa-miR-582-5p	-0.216	0.021309	0.249	hsa-miR-326
-0.207	0.0069353	0.0774	hsa-miR-142-5p	-0.213	0.0229003	0.26	hsa-miR-30e-3p
0.206	0.007109	0.0774	hsa-miR-331-3p	-0.212	0.0240042	0.261	hsa-miR-181c-5p
-0.206	0.0072176	0.0774	hsa-miR-425-3p	-0.211	0.0243525	0.261	hsa-miR-151-3p
0.205	0.0075385	0.0786	hur_6	-0.207	0.0271023	0.283	hsa-miR-30c-2-3p
-0.204	0.0078614	0.0799	hsa-miR-214-3p	-0.206	0.0282946	0.287	dmr_3
0.2	0.008842	0.0843	hsa-miR-125a-5p	-0.204	0.0292634	0.29	hsa-miR-1271-5p
0.2	0.0091506	0.0843	hsa-miR-30c-5p	0.2	0.032661	0.315	hsa-miR-214-3p
-0.2	0.0091566	0.0843	hsa-miR-1246	-0.199	0.0337918	0.318	hsa-miR-30b-3p
-0.2	0.0091705	0.0843	hsa-miR-199a-3p	-0.198	0.0353433	0.325	hsa-miR-3065-5p
0.197	0.0100353	0.0893	hsa-miR-30b-3p	0.196	0.0366227	0.326	hsa-miR-320c
-0.197	0.0101837	0.0893	hsa-miR-144-3p	-0.195	0.0376419	0.326	dmr_6
-0.196	0.0104942	0.09	hsa-miR-100-5p	-0.195	0.0380465	0.326	hsa-miR-27a
0.193	0.0117325	0.0985	hsa-miR-218-5p	0.194	0.0392238	0.329	hsa-miR-185-5p
0.192	0.0122758	0.099	hsa-miR-130a-3p	-0.19	0.043302	0.356	hsa-miR-542-5p
0.192	0.0123072	0.099	hsa-miR-151a-5p	-0.189	0.0443359	0.357	hsa-miR-1288-3p
0.191	0.0125717	0.099	hsa-miR-197-3p	0.188	0.0453903	0.358	hsa-miR-16-5p
0.186	0.0153498	0.119	hsa-miR-326	0.186	0.0476306	0.368	hsa-miR-16-2-3p
-0.185	0.016064	0.12	hsa-miR-19a-3p				
-0.184	0.0161451	0.12	hsa-miR-376a-3p				
-0.183	0.0168351	0.121	hsa-miR-495-3p				
0.183	0.0169747	0.121	hsv1-miR-H8-5p				
0.182	0.0178023	0.123	hsa-miR-4270				
-0.182	0.0179098	0.123	hsa-miR-584-5p				
0.181	0.0181485	0.123	hsa-miR-187-5p				
0.18	0.0186406	0.124	hsa-miR-1181				
0.18	0.0190584	0.125	hsa-miR-224-5p				
-0.179	0.0195736	0.126	hsa-miR-146b-5p				
0.178	0.0199528	0.126	hsa-miR-23a-3p				
0.176	0.0219107	0.136	hsa-miR-4322				
-0.175	0.0229059	0.14	hsa-miR-3651				
0.174	0.0232032	0.14	hsa-miR-99b-5p				
-0.173	0.0241515	0.141	hsa-miR-136-3p				
-0.173	0.0242629	0.141	hsa-miR-125b-5p				
0.172	0.0248808	0.141	hsv2-miR-H24				
0.172	0.0250556	0.141	hsa-miR-34c-5p				
-0.172	0.0252105	0.141	hsa-miR-16-5p				
0.171	0.0257163	0.142	hsa-miR-34b-5p				
0.17	0.0262983	0.143	hsa-miR-574-3p				
-0.17	0.0268006	0.144	hsa-miR-34a-5p				
-0.169	0.0280249	0.148	hsa-miR-4284				
-0.167	0.0300203	0.155	hsa-miR-140-5p				
0.167	0.0300276	0.155	hsa-miR-1249-3p				
0.165	0.0316558	0.161	hsa-miR-1226-5p				
0.164	0.0324755	0.163	hsa-miR-30d-3p				
0.161	0.035615	0.175	hsa-miR-296-5p				
0.161	0.0359346	0.175	hsa-miR-452-5p				
-0.161	0.036357	0.175	hsa-miR-199b-5p				
0.16	0.0376937	0.18	hsa-miR-26a-5p				
0.157	0.0408673	0.19	hsa-miR-141-3p				
-0.157	0.0409179	0.19	hsa-miR-376c-3p				
0.156	0.0428897	0.197	hsa-miR-221-3p				
0.154	0.0443135	0.199	hsv2-miR-H22				
0.154	0.0447422	0.199	hsa-miR-33a-5p				
-0.154	0.0448448	0.199	hsa-miR-451a				
-0.152	0.047776	0.21	hsa-miR-15a-5p				
0.151	0.0491452	0.213	hsa-miR-3188				

Supplemental Table 2:
Characteristics of the LGRC cohort (Discovery and Validation cohorts)

	Discovery cohort			Validation cohort		
	**No COPD**	**GOLD I, II**	**GOLD III, IV**	**No COPD**	**GOLD I, II**	**GOLD III, IV**
**Subjects,** N	28	36	20	50	14	24
**Sex,** N (%)						
Female	15 (54%)	17 (47%)	11 (55%)	29 (58%)	7 (50%)	14 (58%)
Male	13 (46%)	19 (53%)	9 (45%)	21 (42%)	7 (50%)	10 (42%)
**Self-reported race/ethnicity,** N (%)						
White	24 (86%)	35 (97%)	19 (95%)	49 (98%)	13 (93%)	23 (96%)
African American	1 (4%)	1 (3%)	0 (0%)	1 (2%)	1 (7%)	1 (4%)
Hispanic	1 (4%)	0 (0%)	0 (0%)	0 (0%)	0 (0%)	0 (0%)
Asian or Pacific Islander	1 (4%)	0 (0%)	0 (0%)	0 (0%)	0 (0%)	0 (0%)
Multiracial, multi-ethnic, or did not disclose	1 (4%)	0 (0%)	1 (5%)	0 (0%)	0 (0%)	0 (0%)
**Age,** median (IQR)	64 (56-73)	70 (65-74)	64 (57-70)	66 (60-72)	64 (60-73)	62 (51-67)
**FEV_1 % predicted,** median (IQR)	97 (87-105)	62 (55-73) [1]	25 (22-33) [1]	91 (85-101)	59 (50-72)	22 (16-28)
**Radiographic emphysema %,** median (IQR)	0.14 (0.09-0.45) [25]	2.55 (0.78-11.20)	25.50 (14.90-37.80) [2]	0.26 (0.14-0.54) [23]	4.45 (0.87-9.00)	25.90 (18.00-32.10)
**Smoking status,** N (%)						
Former	15 (57%)	31 (86%)	18 (90%)	30 (60%)	14 (100%)	23 (96%)
Never	8 (29%)	1 (3%)	1 (5%)	14 (28%)	0 (0%)	0 (0%)
Current	0 (0%)	4 (11%)	1 (5%)	1 (2%)	0 (0%)	1 (4%)
Missing data	4 (14%)	0 (0%)	0 (0%)	5 (10%)	0 (0%)	0 (0%)
**Pack-year,** median (IQR)	7 (0 - 26) [6]	60 (31 - 115)	50 (42 - 80)	17 (0 - 47) [5]	50 (40 - 80)	40 (21 - 59) [1]

IQR, interquartile range
[] represents missing data.

Supplemental Table 3. Characteristics of the Confirmatory cohort			
	**No COPD**	**GOLD I, II**	**GOLD III, IV**
**Subjects,** N	28	35	24
**Sex,** N (%)			
Female	11 (39%)	14 (40%)	11 (46%)
Male	17 (61%)	21 (60%)	13 (54%)
**Self-reported race/ethnicity**, N (%)			
White	28 (100%)	35 (100%)	22 (92%)
African American	0 (0%)	0 (0%)	2 (8%)
Hispanic	0 (0%)	0 (0%)	0 (0%)
Asian or Pacific Islander	0 (0%)	0 (0%)	0 (0%)
Multiracial, multi-ethnic, or did not disclose	0 (0%)	0 (0%)	0 (0%)
**Age,** mean ± SD	65 ± 13	71 ± 9	58 ± 12
**FEV_1 % predicted,** mean ± SD	96 ± 14	69 ± 15	26 ± 6
**Radiographic emphysema %,** median (IQR)	0.25 (0.13-2.26) [12]	3.7 (1.00-7.30)	25.80 (9.10-36.40)
**Smoking status,** N (%)			
Former	19 (68%)	32 (91%)	24 (100%)
Never	7 (25%)	2 (6%)	0 (0%)
Current	0 (0%)	1 (3%)	0 (0%)
Missing data	2 (7%)	0 (0%)	0 (0%)
**Pack-years,** median (IQR)	12 (0-53) [13]	50 (37-77)	42 (20-65) [1]

SD, standard deviation
IQR, interquartile range.
[] represents missing data.

Supplemental Table 4. Characteristics of the COSMIC cohort				
	**Never smokers**	**Smokers without COPD**	**Smokers with COPD (GOLD I)**	**Smokers with COPD (GOLD II)**
**Subjects**, N	22	10	18	13
**Sex**, N (%)				
Female	11 (50%)	3 (30%)	10 (56%)	5 (39%)
Male	11 (50%)	7 (70%)	8 (44%)	8 (62%)
**Age,** median (IQR)	59 (47-62)	55 (50-62)	61 (54-64)	63 (59-65)
**FEV_1 % predicted**, mean ± SD	116 ± 14	107 ± 10	87 ± 5	69 ± 9
**Smoking status**, N (%)				
Former	0 (0%)	0 (0%)	6 (33%)	5 (39%)
Never	22 (100%)	0 (0%)	0 (0%)	0 (0%)
Current	0 (0%)	10 (100%)	12 (67%)	8 (62%)
Missing	0 (0%)	0 (0%)	0 (0%)	0 (0%)
**Pack-years,** mean ± SD	-	35 ± 7	36 ± 9	42 ± 15

SD, standard deviation.
IQR, interquartile range.

**Supplemental Table 5. Characteristics of study patients for BIM western blots**		
	**GOLD I**	**GOLD III/IV**
**Subjects**, N	6	9
**Sex,** N (%)		
Female	2 (33%)	4 (44%)
Male	4 (67%)	5 (56%)
**Self-reported race/ethnicity**, N (%)		
White	5 (83%)	8 (89%)
African American	1 (17%)	1 (11%)
Hispanic	0 (0%)	0 (0%)
Asian or Pacific Islander	0 (0%)	0 (0%)
Multiracial, multi-ethnic, or did not disclose	0 (0%)	0 (0%)
**Age,** median (IQR)	71 (68-82)	59 (56-65)
**FEV_1 % predicted,** median (IQR)	93 (79-100)	21 (19-25)
**Smoking status**, N (%)		
Former	6 (100%)	9 (100%)
Never	0 (0%)	0 (0%)
Current	0 (0%)	0 (0%)
Missing	0 (0%)	0 (0%)
**Pack-years**, median (IQR)	38 (20-69) [1]	38 (30-54)

IQR, interquartile range.
[] represents missing data.

Supplemental Table 6. Characteristics of study patients for BRCA1 AQUA.		
	**No COPD**	**COPD**
**Subjects**, N	9	16
**Sex,** N (%)		
Female	4 (44%)	4 (25%)
Male	5 (56%)	12 (75%)
**Self-reported race/ethnicity,** N (%)		
White	9 (100%)	14 (88%)
African American	0 (0%)	2 (13%)
Hispanic	0 (0%)	0 (0%)
Asian or Pacific Islander	0 (0%)	0 (0%)
Multiracial, multi-ethnic, or did not disclose	0 (0%)	0 (0%)
**Age,** mean ± SD	62 ± 14	63 ± 8
**FEV_1 % predicted,** median (IQR)	89 (82-94)	25 (22-35)
**Radiographic emphysema %,** median (IQR)	0.03 (0–0.19)	30.20 (17.20–43.10)
**Smoking status, N (%)**		
Former	8 (89%)	16 (100%)
Never	1 (11%)	0 (0%)
Current	0 (0%)	0 (0%)
Missing	0 (0%)	0 (0%)
**Pack-years,** median (IQR)	19 (2-46) [1]	43 (32-74)

SD, standard deviation
IQR, interquartile range
[] represents missing data.

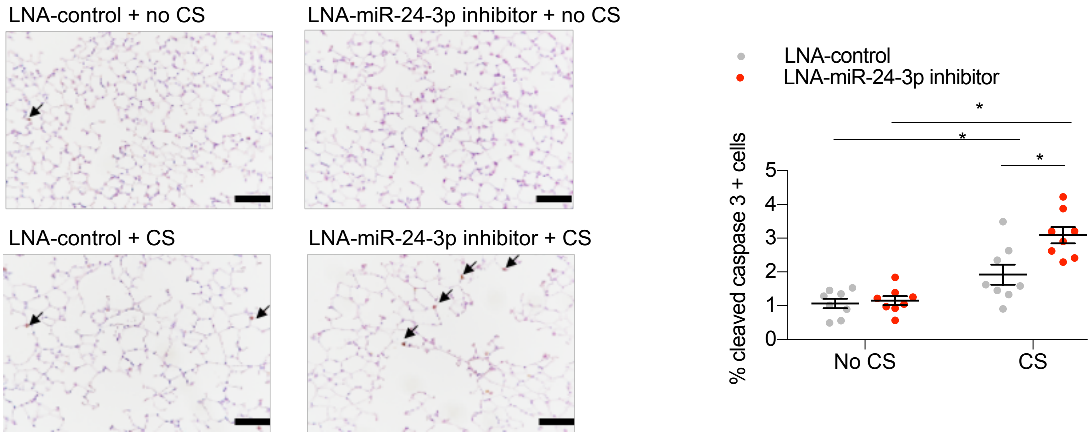

Supplemental Figure 1. (*left*) Representative images of peripheral lung tissue following immunohistochemistry for cleaved caspase 3 in mice treated with locked nucleic acid (LNA)-miR-24-3p inhibitor or LNA-control ± exposure to cigarette smoke (CS). Black scale bar = 200 μM. Arrows demonstrate cleaved caspase 3 positive cells. *(right)* Quantification of cleaved caspase-3 in lungs from mice treated with LNA-miR-24-3p inhibitor vs. LNA-control ± CS exposure. n=8/group. Error bars represent mean ± SEM., *P<0.05, ordinary one-way ANOVA correcting for multiple comparisons using 2-stage linear step-up procedure of Benjamini, Krieger, and Yekutieli.

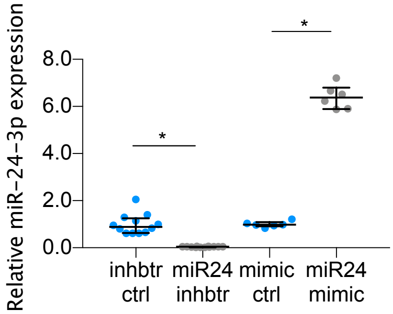

Supplemental Figure 2. Relative miR-24-3p expression (ΔΔCt miR-24-3p/RNU48) as assessed by RT-PCR following treatment with miR-24-3p mimic vs. mimic control (n=6/group) or miR-24-3p inhibitor vs. inhibitor control (n=12/group) Error bars represent median ± interquartile range. *P<0.05 Mann-Whitney.

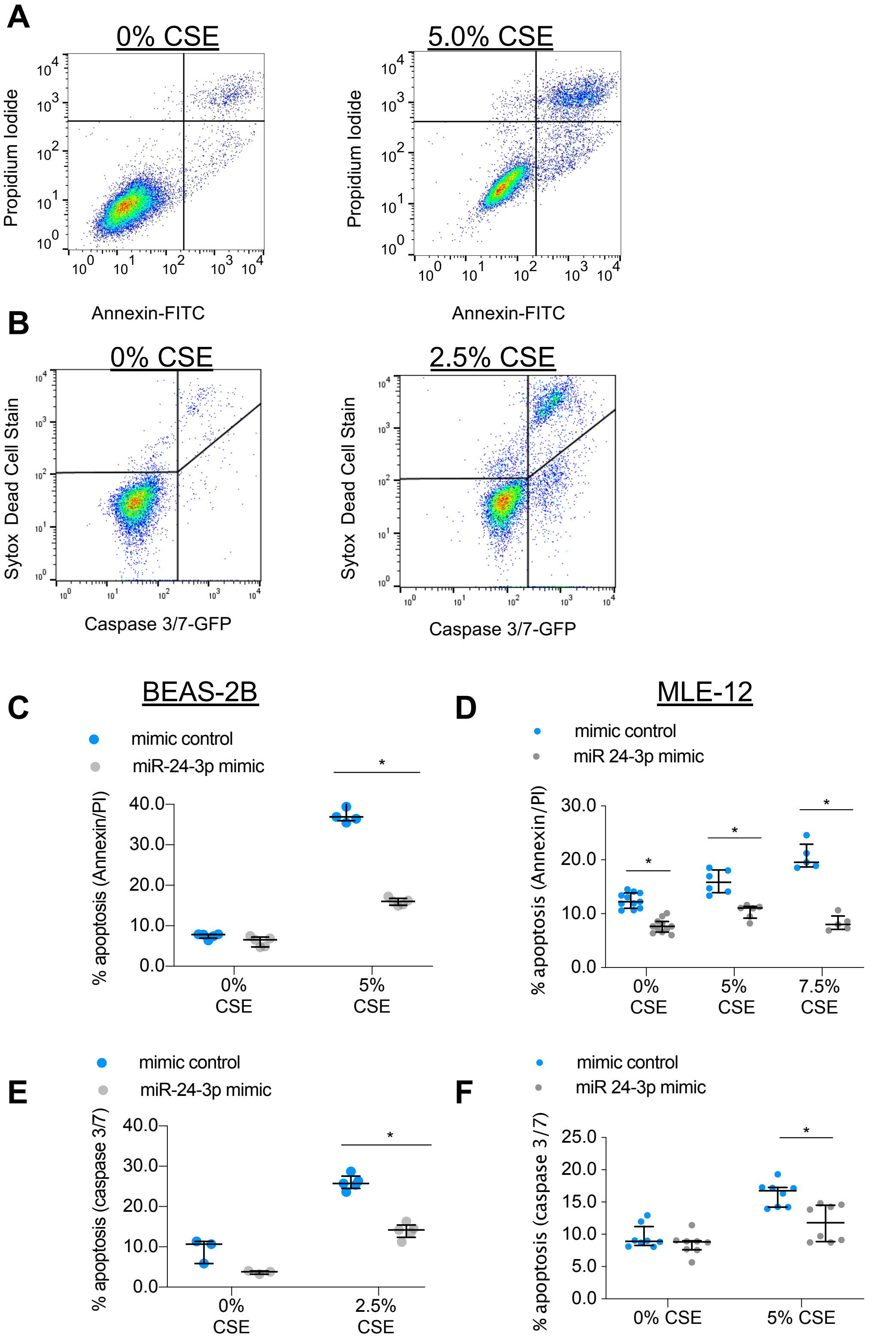

Supplemental Figure 3. A) Flow cytometric detection of Propidium Iodide (y-axis) and Annexin V (x-axis) in cells exposed to 0% cigarette smoke extract (CSE) (*left*) or 5% CSE (*right*) **B)** Flow cytometric detection of Sytox AAdvanced(y-axis) and caspase 3/7 (x-axis) in cells exposed to 0% CSE (*left*) or 2.5% CSE (*right*). **C,D)** Percent apoptotic cells determined by Annexin/PI staining in miR-24-3p mimic vs. mimic control treated BEAS-2B cells (n=5/group) and MLE-12 cells (n=5-11/group) exposed to indicated percentages of CSE. **E,F)** Percent apoptotic cells determined by caspase 3/7 and Sytox AAdvanced in miR-24-3p mimic and mimic control treated BEAS-2B cells (*left*) (n=3-4/group) and MLE-12 cells (*right*) (n=8/group) exposed to indicated percentages of CSE. Error bars represent median ± interquartile range. *P<0.05 Kruskal-Wallis correcting for multiple comparisons using 2-stage linear step-up procedure of Benjamini, Krieger, and Yekutieli.

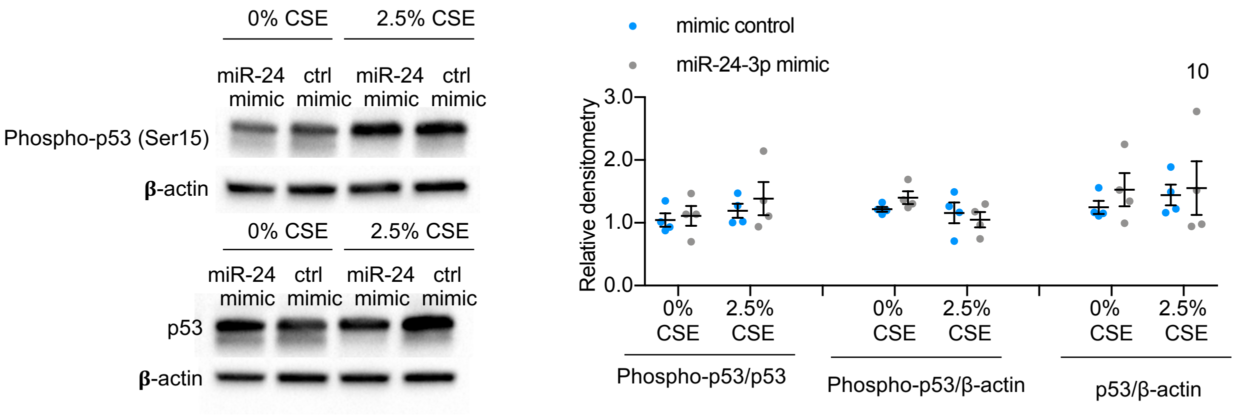

Supplemental Figure 4. (*left*) Sample immunoblotting for phosphorylated p53 (Serine 15), p53, and β-actin performed on lysates of BEAS-2B cells treated with miR-24-3p mimic, mimic control, miR-24-3p inhibitor, or inhibitor control ± cigarette smoke extract (CSE). (*right)* Relative densitometry of phosphorylated p53 (Serine 15), p53, and β-actin. n=4/group. Error bars represent median ± interquartile range.

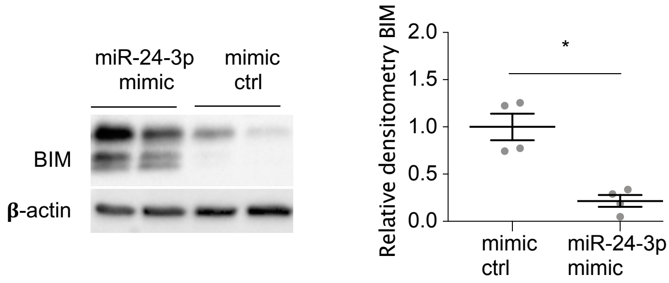

Supplemental Figure 5. Sample immunoblotting and relative densitometry of BIM in MLE-12 cells following treatment with miR-24-3p mimic or mimic control (ctrl) n=4/group. Error bars represent median ± interquartile range. *P<0.05 using Mann-Whitney.

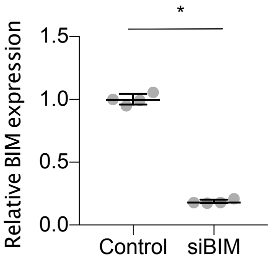

Supplemental Figure 6. Relative BIM expression in BEAS-2B cells following treatment with scrambled control or silencing RNA targeted against BIM (siBIM). n=4/group. Error bars represent median ± interquartile range. *P<0.05 using Mann-Whitney.

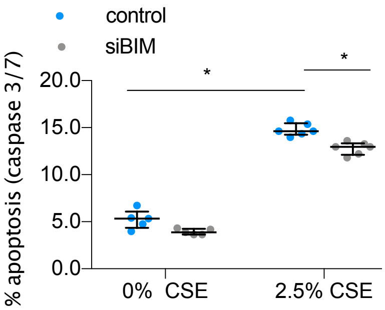

Supplemental Figure 7. Percent apoptotic cells determined by flow cytometry for caspase 3/7 and Sytox AAdvanced in BEAS-2B treated with scrambled control or silencing RNA targeted against BIM (siBIM) and exposed to 0% or 2.5% CSE. n=5-6/group. Error bars represent median ± interquartile range. *P<0.05, Kruskal-Wallis correcting for multiple comparisons using 2-stage linear step-up procedure of Benjamini, Krieger, and Yekutieli.

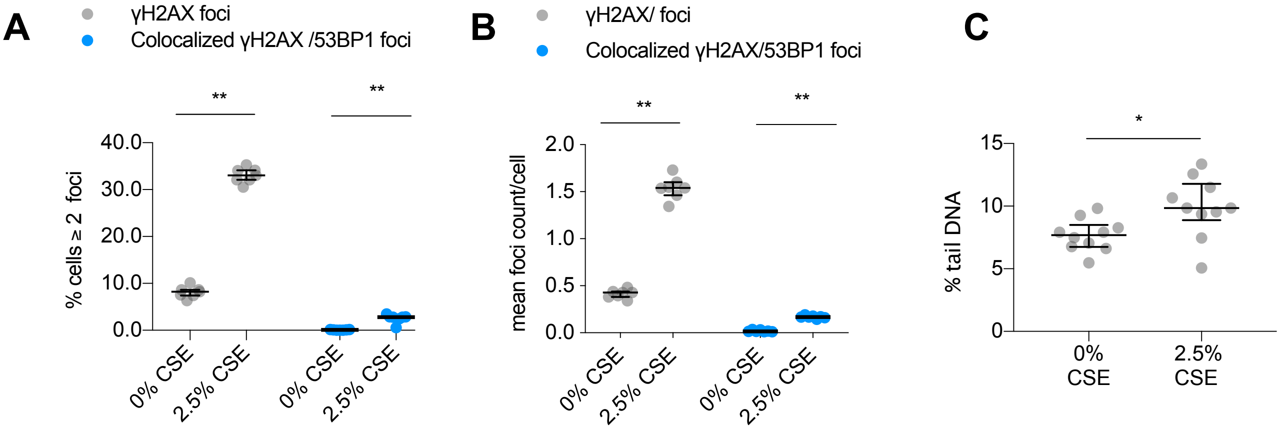

Supplemental Figure 8. A) Number of **γ**H2AX foci (grey) or colocalized **γ**H2AX and 53BP1 foci (blue) in BEAS2B cells exposed to 2.5% CSE for 24 hours. n=7/group. **B)** Percent of BEAS2B cells with ≧ 2 **γ**H2AX foci (grey) or colocalized **γ**H2AX and 53BP1 foci (blue) over time. n=7/group. **C)** Results of Comet assay in BEAS2B cells exposed to 0% or 2.5% cigarette smoke extract (CSE) for 24 hours. Percent tail DNA reflects increased DNA damage. n=10/group. Error bars represent median ± interquartile range. **P<0.001, *P<0.05 Mann-Whitney.

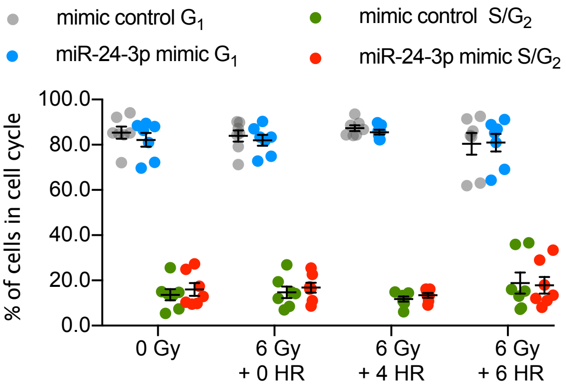

Supplemental Figure 9. Cell cycle phase in primary human airway epithelial cells as estimated by DAPI staining. n=7/group. Error bars represent median ± interquartile range.

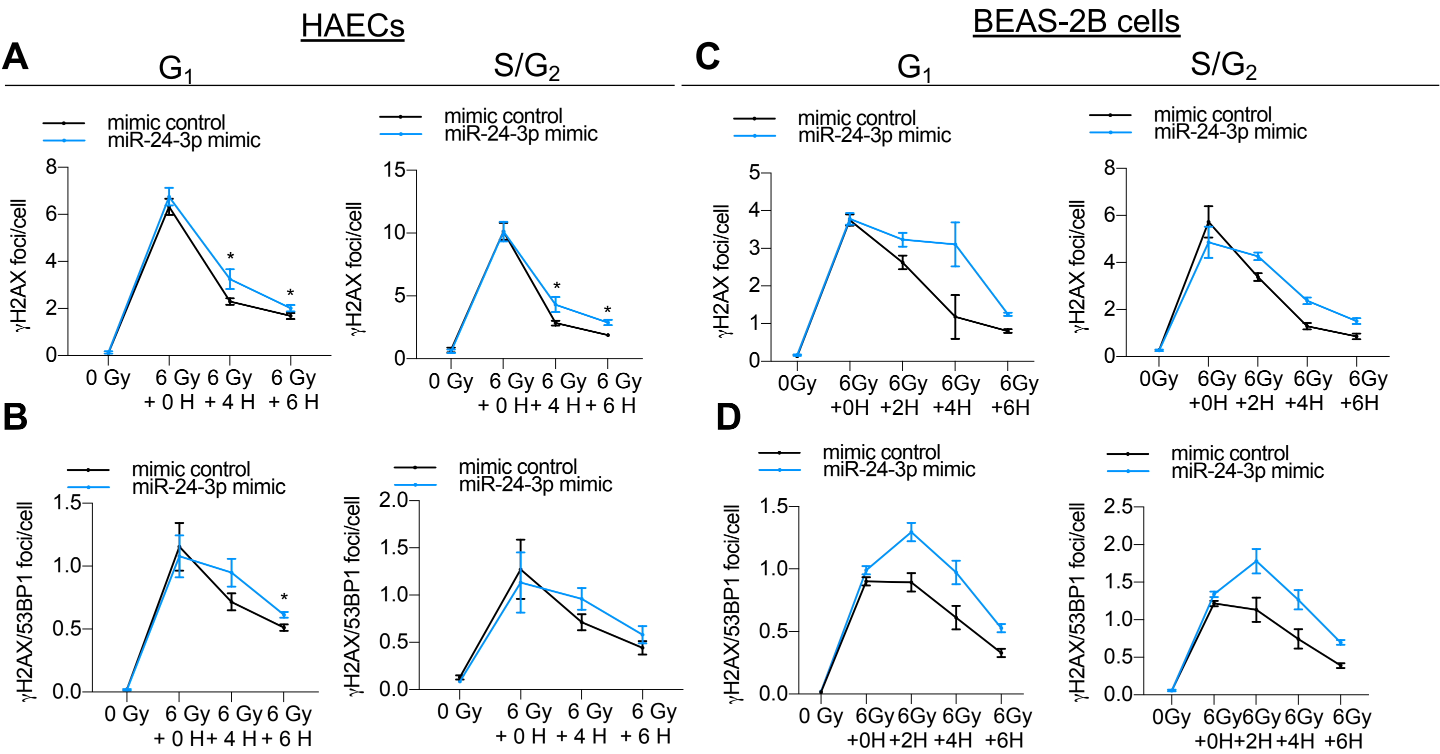

Supplemental Figure 10. A-D) Primary human airway epithelial cells (HAECs) or BEAS-2B (exposed to 0 Grey (Gy) ionizing radiation or 6 Gy with 0-6 hours (H) of recovery to assess kinetics of DNA repair **A**) Number of **γ**H2AX foci in HAECs over time in G_1 or S/G_2 phase of cell cycle. **B)** Number of colocalized **γ**H2AX and 53BP1 foci in HAECs over time in G_1 or S/G_2 of cell cycle **C**) Number of **γ**H2AX foci in BEAS-2B over time in G_1 or S/G_2 phase of cell cycle. **D)** number of colocalized **γ**H2AX and 53BP1 foci in BEAS-2B over time in G_1 or S/G_2 of cell cycle. Error bars represent mean ± SEM (**A, B**) or median ± interquartile range (**C, D**). *$P<0.05$ ordinary one-way ANOVA (**A,B)** or Kruskal-Wallis (**C,D**) correcting for multiple comparisons using 2-stage linear step-up procedure of Benjamini, Krieger, and Yekutieli.

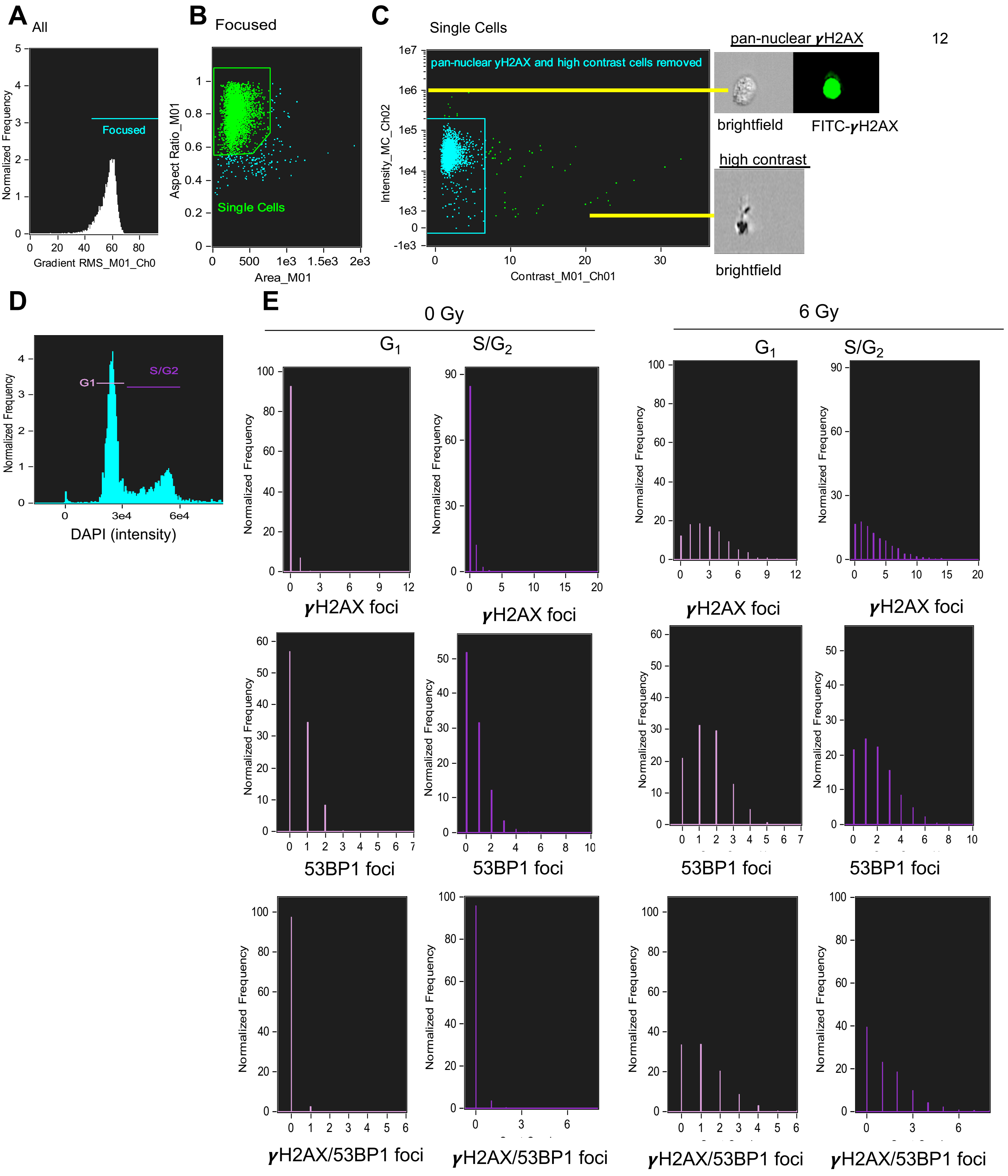

Supplemental Figure 11. A) We used the Gradient RMS feature in the brightfield channel, which indicate sharpness of an image, to identify cells in focus. **B**) We used the area and aspect ratio features in the brightfield channel to identify single cells and remove doublets and debris. **C**) We use the intensity feature in channel 2 to identify pan nuclear **γ**H2AX staining and the contrast feature in the brightfield channel to identify high contrast cells. Both features are suggestive of dead/dying cells. **D**) Cell cycle was estimated from DNA staining with DAPI. **E**) Automated counting of foci of **γ**H2AX , 53BP1, and combined **γ**H2AX/53BP1 was conducted using Imagine software. A distribution of these foci in G_1 and S/G_2 phase following 0 Grey (Gy) or 6 Gy of radiation are shown.

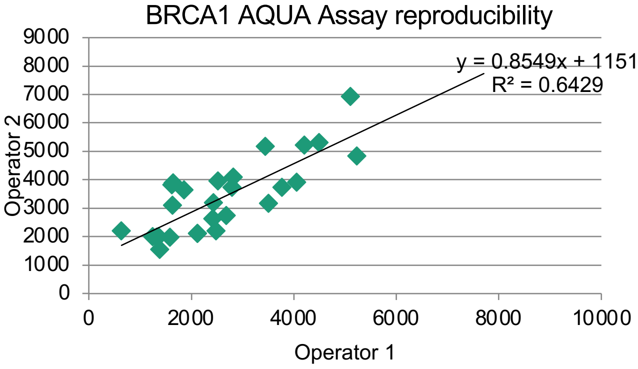

Supplemental Figure 12. To confirm reliability of the BRCA1 AQUA technique, the assay was performed by two independent operators on two separate days. n= 23.

Supplemental Methods:

Lung Genomics Research Consortium (LGRC) cohort: RNA was extracted from flash frozen lung samples using the QIAcube system (Qiagen) with the miRNeasy kit. RNA quality was determined using a Bioanalyzer 2100 (Agilent Technologies) with a RNA integrity number greater than 7.0 as the criterion for acceptable quality. Expression profiles of mRNA and microRNA were obtained using the Agilent Whole Human Genome and Agilent Human microRNA microarrays respectively (Agilent Technologies) and data has been previously deposited in the Gene Expression Omnibus (GSE47460 and GSE72967). We applied cyclic loess normalization for mRNAs and we applied quantile normalization microRNAs. We filtered the microRNAs probes based on a median expression of < 1.7, to obtain 15,261 genes and 526 microRNAs.

Clinical & Systems Medicine Investigations of Smoking-related Chronic Obstructive Pulmonary Disease (COSMIC) cohort: RNA samples were processed on the Agilent 2100 Bioanalyzer small RNA electrophoresis program (Agilent technologies) and hybridized to 1-color to 1-color Agilent custom UCSF microRNA v3.5 multi-species 8x15K Ink-jet arrays (Agilent Technologies).

Quantitative real time-PCR: RNA extraction methods from human samples are described above. RNA was isolated from *in vitro* experiments using the miRNeasy® Mini Kit (Qiagen). For mRNA quantification, cDNA was produced using iScript® cDNA Synthesis Kit (Bio-Rad) and quantitative RT-PCR was performed using SsoFast™ EvaGreen® Supermix with Low ROX (Bio-Rad). For microRNA quantification, cDNA was produced using the Applied Biosystems® TaqMan® MicroRNA Reverse Transcription Kit (Thermo Scientific) with specific primers for miR-24-3p, RNU-48, or snoRNA 202. RT-PCR was performed using Applied Biosystems® TaqMan® Universal PCR Master Mix II (2X) No AmpErase™ UNG (Thermo Fisher Scientific) using the Applied Biosystem's 7500 Real-Time PCR System. TaqMan microRNA primers were purchased from Thermo Fisher Scientific while mRNA primers were purchased from the Yale Keck Synthesis Facility. Primers include:
human hsa-miR-24 and mouse mmu-miR-24 – UGGCUCAGUUCAGCAGGAACAG ; human RNU48 – GATGACCCCAGGTAACTCTGAGTGTGTCGCTGATGCCATCACCGCAGCGCTCTGACC; mouse snoRNA202- GCTGTACTGACTTGATGAAAGTACTTTTGAACCCTTTTCCATCTGATG; Human *BRCA1* forward 5'-GGCTATCCTCTCAGAGTGACATTT-3' and reverse 5' GCTTTATCAGGTTATGTTGCATGGT-3';

Human *BIM* forward 5'-TGGCAAAGCAACCTTCTGATG-3' and reverse 5'-GCAGGCTGCAATTGTCTACCT-3'; Human *18S* forward 5'-CCATCCAATCGGTAGTAGCG-3' and reverse 5'-GTAACCCGTTGAACCCCATT-3'.

AMNIS flow cytometry: Cells were analyzed with AMNIS® imaging flow-cytometer (MilliporeSigma). At least 1000 cells were captured per condition at 40x magnification with extended depth of field. Data was analyzed using IDEAS™ 6.2 imaging flow cytometry software. First, single round-cells were gated according to their aspect ratio (>0.6) area (arbitrary units) in the brightfield channel. Second, well-focused cells were selected in the brightfield channel using the gradient similarity feature. High contrast cells and cells with bright pan-nuclear γ-H2AX staining (both representing dead or apoptotic cells) were excluded. Gating on the fluorescence channel with the nuclear stain was used to assess cell cycle phase (G_1 and S/G_2). To identify γ-H2AX, 53BP1, and co-localized γ-H2AX and 53BP1 foci, we utilized "Spot", "Peak", and "Intensity" imaging masks using IDEAS 6.2 which were counted in an automated fashion using imaging flow cytometry software (MilliporeSigma).

Mouse lung sample collection, physiologic, and morphometric assessment of lungs: Mice received an intraperitoneal urethane injection (0.1 mg/kg, Sigma, U2500) to avoid hypoventilation, the trachea was cannulated, and the mice were paralyzed with pancuronium bromide (0.1 mg/kg; i.p., Sigma, P1918). The mouse was connected to a SCIREQ flexiVent (SCIREQ), lungs were ventilated at 10 cc/kg, and flexiware software (SCIREQ) was used to assess Pressure-Volume curves and lung compliance. The cannulated trachea was perfused with two 0.8-ml aliquots of cold saline. The cellular contents and bronchoalveolar lavage (BAL) fluid were separated by centrifugation. Cells were reconstituted in PBS and cell counts were obtained via a Coulter counter (Beckman Coulter). A subset of lung samples was flash frozen in liquid nitrogen and others were inflated slowly with 1% low-temperature melting agarose at a constant pressure of 25 cm. H&E images were acquired with Images with Nikon DS-Ri2. A minimum of six random fields were evaluated by microscopic projection and the NIH image program; alveolar size was estimated from the mean linear intercept (L_m) of the air space as described previously (1).

1. Sauler M, et al. Macrophage migration inhibitory factor deficiency in chronic obstructive pulmonary disease. *Am J Physiol Lung Cell Mol Physiol.* 2014;306(6):L487-96.

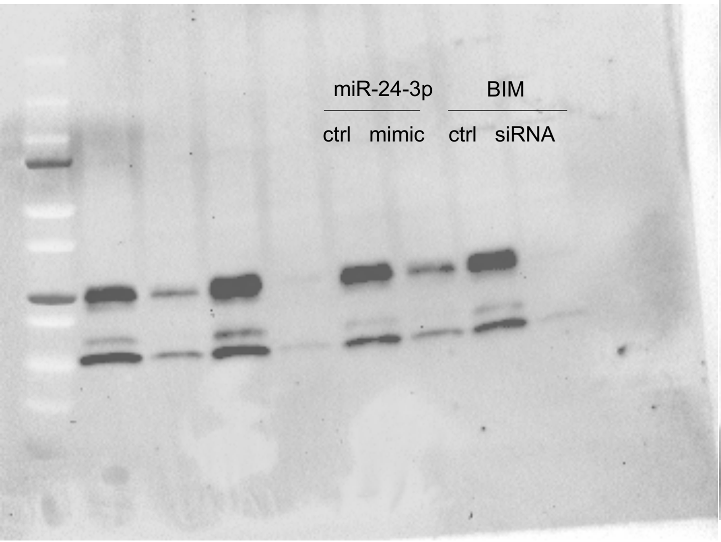

Full unedited gel for Figure 4D - BIM

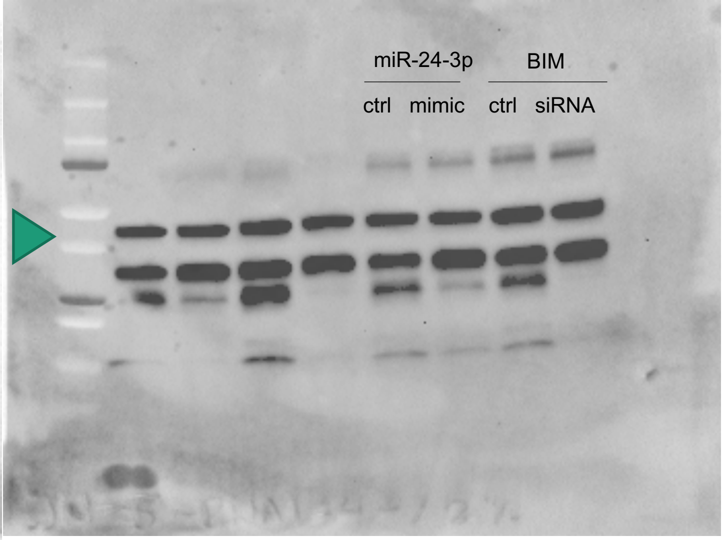

Full unedited gel for Figure 4D - **β**-actin

Lanes represent treatment with :
1) marker
2) miR-24-3p ctrl
3) miR-24-3p mimic
4) BIM siRNA control
5) BIM siRNA
6) Blank
7) miR-24-3p ctrl
8) miR-24-3p mimic
9) BIM siRNA control
10) BIM siRNA

Lanes 6-8 used for manuscript
β-actin labeled (green arrow)

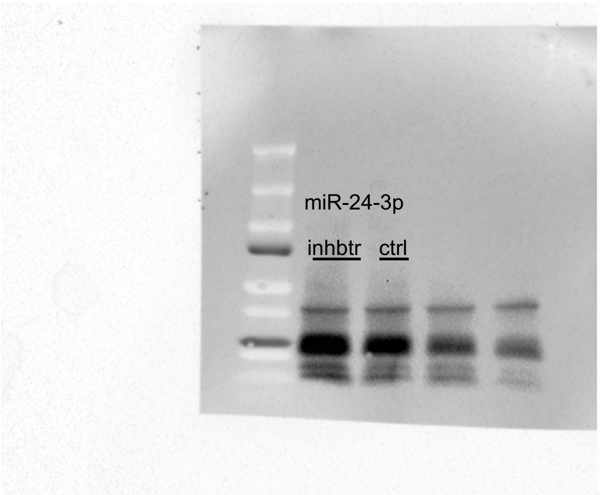

Full unedited Gel for Figure 4D - BIM

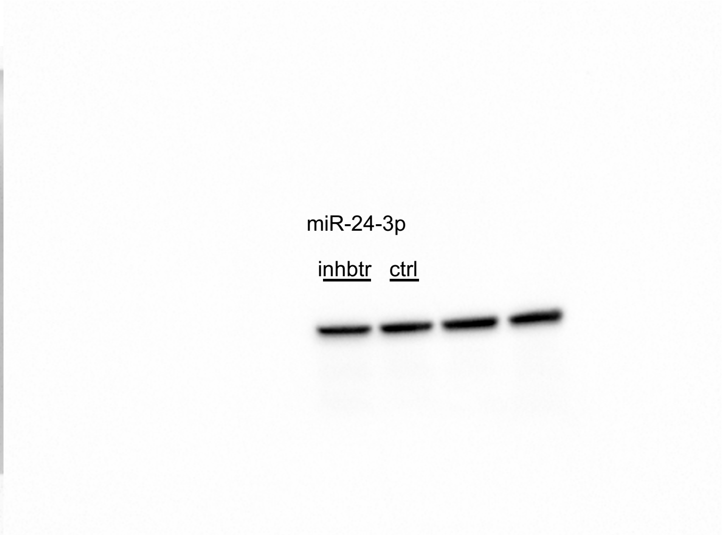

Full unedited Gel for Figure 4D - **β**-actin

Lanes represent treatment with
1) miR-24-3p inhibitor
2) miR-24-3p Inhibitor control
3) miR-24-3p inhibitor + cigarette smoke extract
4) miR-24-3p inhibitor control + cigarette smoke extract

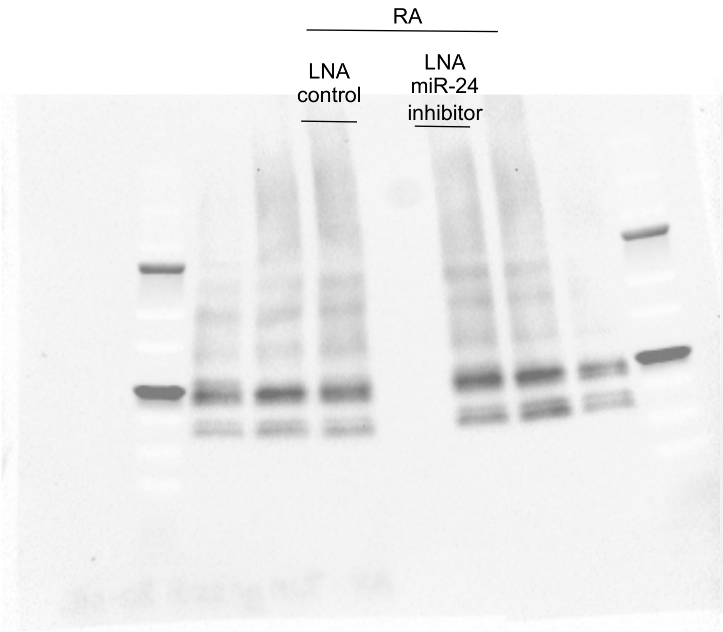

Full unedited gel for Figure 4G - BIM

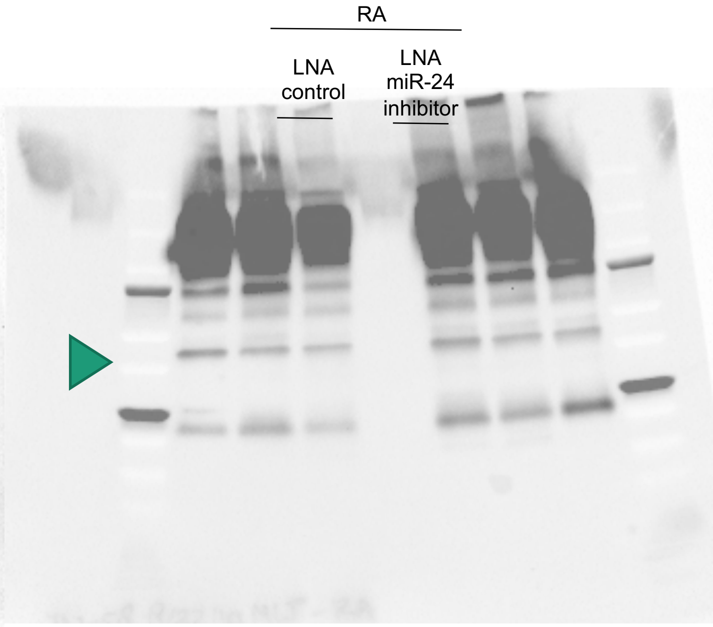

Full unedited gel for Figure 4G - **β**-actin

Lanes represent treatment with:

1) Marker
2) Room air exposed mouse + LNA-control
3) Room air exposed mouse + LNA-control
4) Room air exposed mouse + LNA-control
5) BLANK
6) Room air exposed mouse + LNA-miR-24 inh
7) Room air exposed mouse + LNA-miR-24 inh
8) Room air exposed mouse + LNA-miR-24 inh
9) Marker

Lanes 4-6 used for manuscript
β-actin labeled (green arrow)

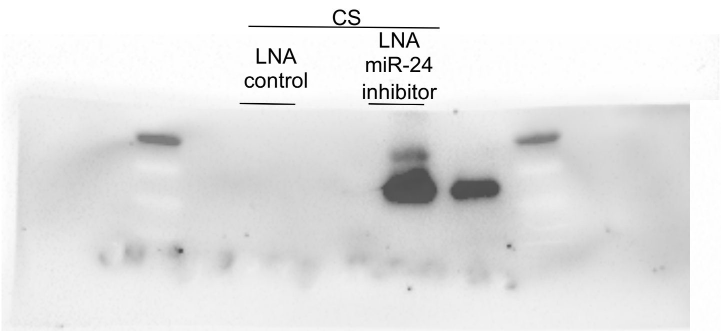

Full unedited gel for Figure 4G - BIM

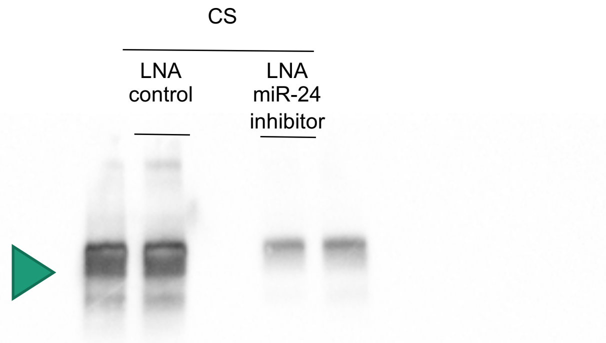

Full unedited gel for Figure 4G - β-actin

Lanes represent:
1) Marker
2) Cigarette smoke exposed mouse + LNA-control
3) Cigarette smoke exposed mouse + LNA-control
4) BLANK
5) Cigarette smoke exposed mouse + LNA-miR-24 inh
6) Cigarette smoke exposed mouse + LNA-miR-24 inh
7) Marker

Lanes 3-5 used for manuscript
β-actin labeled (green arrow)

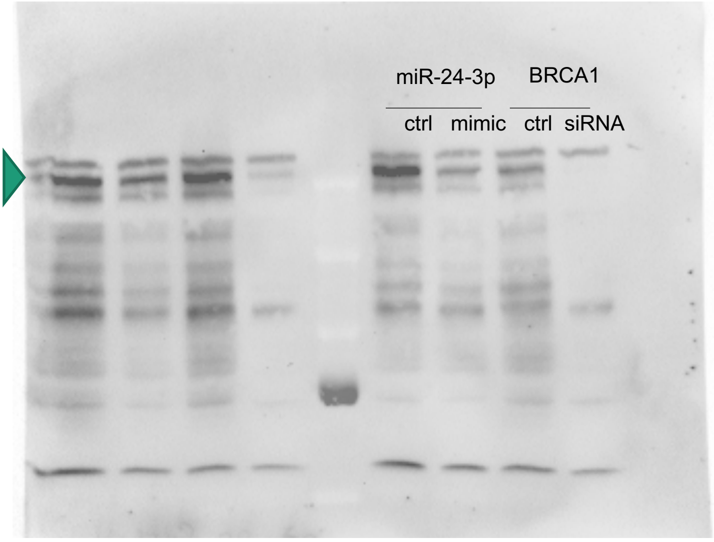

Full unedited gel for Figure 5J – BRCA1

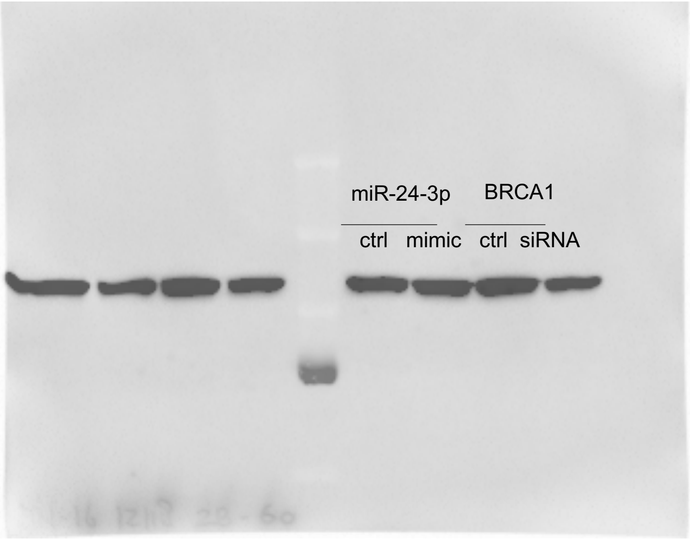

Full unedited gel for Figure 5J - Vinculin

Lanes represent:
1) marker
2) miR-24-3p ctrl
3) miR-24-3p mimic
4) BRCA1 siRNA control
5) BRCA1 siRNA
6) Blank
7) miR-24-3p ctrl
8) miR-24-3p mimic
9) BRCA1 siRNA control
10) BRCA1 siRNA

Lanes 6-8 used for manuscript
Green arrow = BRCA1

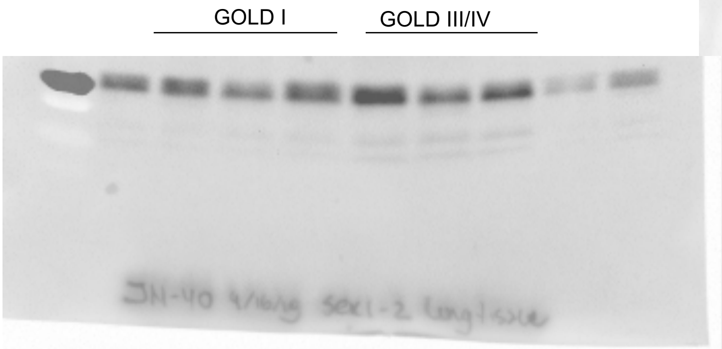

Full unedited gel for Figure 6D – BIM

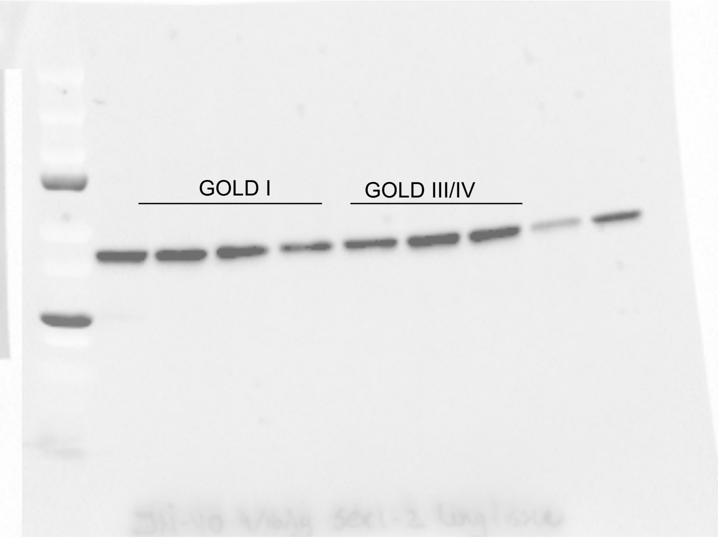

Full unedited gel for Figure 6D - **β**-actin

Lanes represent:
1) marker
2-5) GOLD I COPD
6-10) GOLD III/IV COPD
Lanes 2-7 used for manuscript

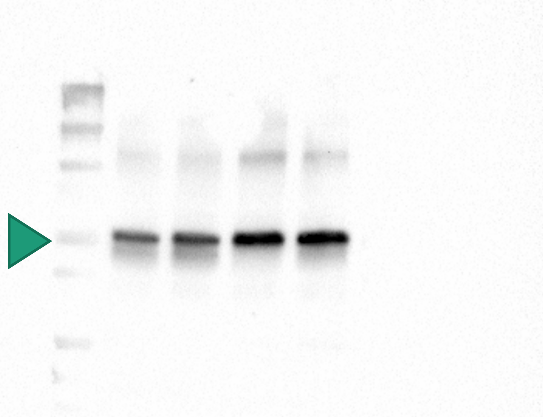

Full unedited get for
Supplemental Figure 4
phospho-p53 (green arrow)

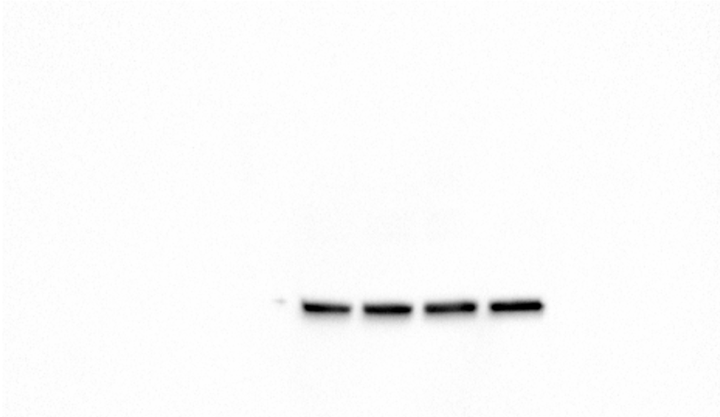

Full unedited gel for
Supplemental Figure 4
β-actin

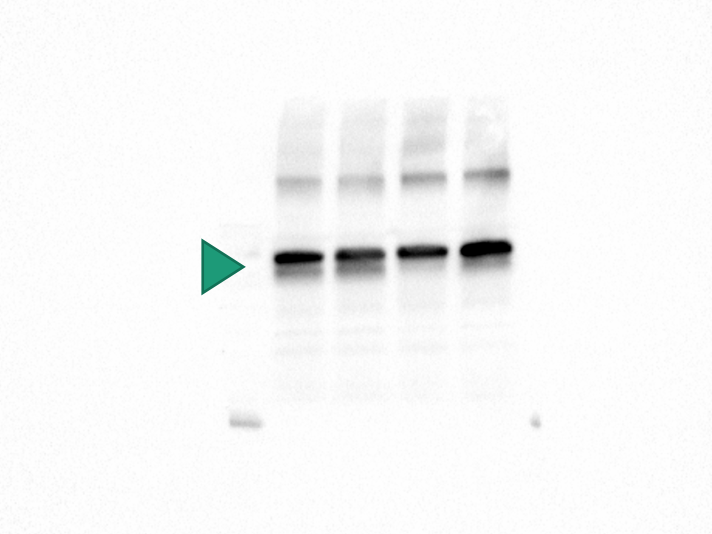

Full unedited gel for
Supplemental Figure 4
P53 (green arrow)

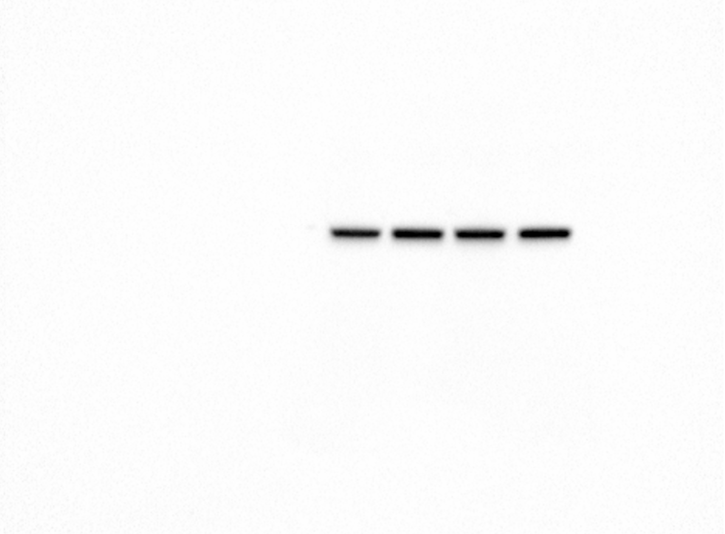

Full unedited gel for
Supplemental Figure 4
β-actin

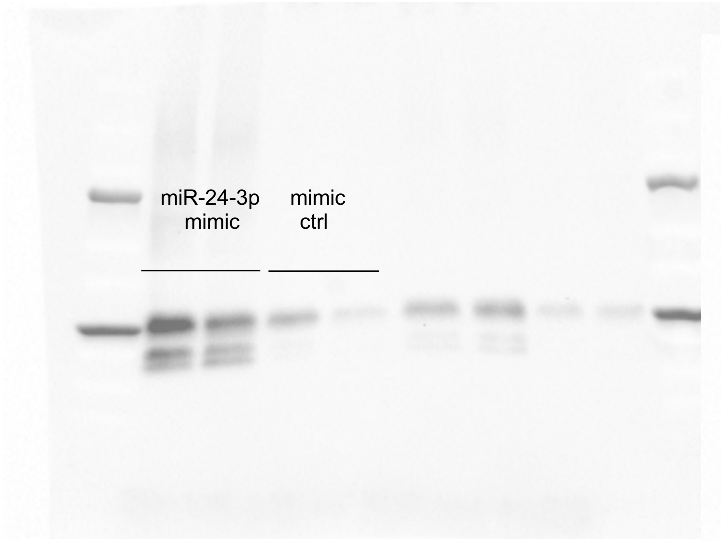

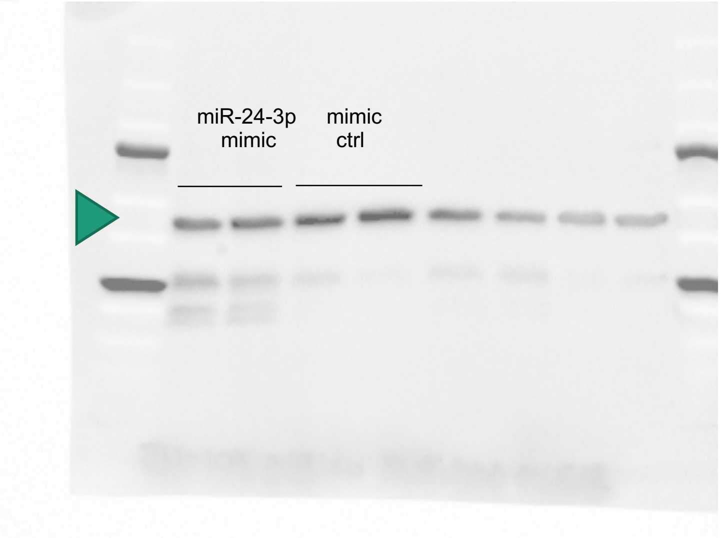

Full unedited gel for Supplemental Figure 5 BIM

Full unedited gel for Supplemental Figure 5 **β**-actin

Lanes represent:
1) marker
2) miR-24-3p mimic
3) miR-24-3p mimic
4) mimic ctrl
5) Mimic ctrl
6) miR-24-3p mimic
7) miR-24-3p mimic
8) miR-24-3p ctrl
9) miR-24-3p ctrl
10 marker

Lanes 2-5 used for manuscript
Green arrow = **β**-actin
